# Electroceutical enhancement of self-compassion training using transcutaneous vagus nerve stimulation: results from a preregistered fully factorial randomized controlled trial

**DOI:** 10.1017/S0033291725101013

**Published:** 2025-08-04

**Authors:** Sunjeev K. Kamboj, Matthew Peniket, Jessica Norman, Rosalind Robshaw, Amit Soni-Tricker, Caroline Falconer, Paul Gilbert, Louise Simeonov

**Affiliations:** 1 Clinical Psychopharmacology Unit, University College London, London, UK; 2 Research Department of Clinical, Educational and Health Psychology, University College London, London, UK; 3 School of Psychology, College of Health, Psychology and Social Care, University of Derby, Derby, UK

**Keywords:** compassion, mindfulness, transcutaneous vagus nerve stimulation, neurostimulation, compassion-focused therapy, attentional bias, heart rate variability

## Abstract

**Background:**

Physiological signals conveyed by the vagus nerve may generate quiescent psychological states conducive to contemplative practices. This suggests that vagal neurostimulation could interact with contemplative psychotherapies (e.g. mindfulness and compassion-based interventions) to augment their efficacy.

**Methods:**

In a fully factorial experimental trial, healthy adults (*n* = 120) were randomized to transcutaneous vagus nerve stimulation (tVNS) *plus* Self-Compassion-Mental-Imagery Training (SC-MIT) *or* alternative factorial combinations of *stimulation* (tVNS or sham) plus *mental imagery training* (MIT: SC-MIT or Control-MIT). Primary outcomes were self-reported state self-compassion, self-criticism, and heart rate variability (HRV). Exploratory outcomes included state mindfulness and oculomotor attentional bias to compassion-expressing faces. Most outcomes were assessed acutely on session 1 at the pre-stimulation (T1), peri-stimulation (T2), and post-MIT + stimulation (T3) timepoints, and after daily stimulation+MIT sessions (eight sessions).

**Results:**

During session 1, a significant Timepoint × Stimulation × MIT interaction (*p* = 0.025) was observed, reflecting a larger acute T1→T3 increase in state self-compassion after tVNS+SC-MIT, with similar rapid effects on state mindfulness. Additionally, significant Session × MIT and Session × Stimulation interactions (*p* ≤ 0.027) on state mindfulness (but not self-compassion) suggested that tVNS+SC-MIT’s effects may accumulate across sessions for some outcomes. By contrast, changes in state self-criticism and compassion-related attentional bias were only moderated by MIT (not stimulation) condition. HRV was unaffected by stimulation or MIT condition.

**Conclusion:**

tVNS augmented the effects of SC-MIT and might, therefore, be a useful strategy for enhancing meditation-based psychotherapies. Our findings also highlight the value of oculomotor attentional metrics as responsive markers of self-compassion training and the continued need for sensitive indices of successful vagal stimulation.

## Introduction

The vagus nerve is the primary conveyor of parasympathetic signals between the brain (stem) and viscera (Butt, Albusoda, Farmer, & Aziz, 2020; Ruffoli et al., 2011). In addition to its established role in synchronizing heart rate and respiration (reflected in the respiratory sinus arrhythmia), the vagus nerve may also regulate cognition (Ridgewell et al., 2021) and motivational-affective states (Neuser et al., 2020) via ascending projections from the nucleus solitarius to limbic and forebrain structures implicated in social-affective regulatory processes (Geller & Porges, 2014). Maladaptations in vagal function contribute to neuropsychiatric conditions that can be treated by directly stimulating the vagus nerve using surgically implanted medical devices. More recently, alternative noninvasive strategies have been developed, and preliminary evidence suggests *transcutaneous vagus nerve stimulation* (tVNS) is efficacious in the treatment of psychological disorders such as depression (Tan et al., 2023) and post-traumatic stress disorder (Gurel et al., 2020). However, although tVNS is inexpensive, well-tolerated, and easily self-administered, its optimized therapeutic use requires a thorough understanding of its neurobiological and psychological mechanisms of action.

Experimental psychological studies suggest that tVNS improves performance across various self-regulatory (De Smet et al., 2021) and social-affiliative domains, including emotion recognition (Zhao et al., 2025), interpersonal cooperation (Oehrn et al., 2022), oxytocin release (Zhu et al., 2022) and even spiritual self-concept (Finisguerra, Crescentini, & Urgesi, 2019). These findings align with long-standing ideas about the vagus nerve’s role in higher-order cognitive-affective and affiliative functions, which are proposed to provide a platform for contemplative mental states (Porges, 2017). One such state – ‘compassion’ – refers to cognitive, affective and behavioral responsivity toward – and motivation to alleviate – the suffering of oneself and others. Through sustained contemplative practice (Galante, Galante, Bekkers, & Gallacher, 2014) and/or favorable developmental conditions (Shiota, Keltner, & John, 2006), compassion can develop into a *trait-like* competency expressed both interpersonally and *intra*-personally (*self*-compassion; Neff & Germer, 2017). ‘Mindfulness’ is similarly a self-regulatory capability (Raugh & Strauss, 2024) and involves adopting an open and accepting attentional stance toward the present-moment contents of the mind. Again, regular practice may establish mindful attention as a trait-like capability (Kiken et al., 2015). Whether as dispositions (Tomlinson, Yousaf, Vittersø, & Jones, 2018) or acquired competencies (Galante et al., 2014), (self-)compassion and mindfulness are associated with positive health outcomes (Millard, Wan, Smith, & Wittkowski, 2023; Wielgosz et al., 2019), and recent innovations in psychotherapy have therefore incorporated strategies for developing these competencies into treatment protocols (Hayes & Hofmann, 2021).

Impediments to developing such self-regulatory abilities, including attachment insecurity and a fear of compassion, can limit the efficacy of psychotherapy but can also be overcome within a psychotherapeutic relationship that appropriately engenders ‘safeness’ (Steindl, Bell, Dixon, & Kirby, 2023). Additionally, however, adjunctive treatments that promote self-regulation, insight, psychological flexibility, and/or neuroplasticity (Sayalı & Barrett, 2023) may also counteract the treatment efficacy-limiting effects of such vulnerabilities. Pharmacotherapies proposed for this purpose have significant limitations (Kamboj et al., 2015; Kamboj et al., 2018; Thomas et al., 2021), whereas adjunctive noninvasive neurostimulation techniques that promote adaptive self-regulation and plasticity (Hays, Rennaker II, & Kilgard, 2023; Noble, Souza, & McIntyre, 2019; Peña, Engineer, & McIntyre, 2013; Ruiz et al., 2023) are highly implementable and have a growing evidence base (e.g. Tan et al., 2023).

We recently outlined a rationale for combining tVNS with a specific cognitive-affective self-regulation intervention, namely self-compassion-mental imagery training (Kamboj, Peniket, & Simeonov, 2023). Drawing on prior work (Di Bello et al., 2020; Porges, 2017; Stellar & Keltner, 2017), we postulated that tVNS might generate physiological conditions conducive to compassionate responding, potentially giving rise to (supra)additivity between tVNS and self-compassion training. If observed, such an enhancement could be clinically significant, even if it is short-lived. For example, early ‘hindrances’ in the form of negative beliefs about an ability to meditate predict a lack of persistence in meditation (Russ, Maruyama, Sease, & Jellema, 2017). On the other hand, early experiences of meditation that challenge such beliefs, for example, when an individual finds they are able to respond to perceived ‘failures’ in maintaining focus on the breath with kindness and a willingness to ‘try again’, may encourage the individual to persist.

### Objectives

The main objective of this early-phase exploratory trial was to investigate whether tVNS would interact with (augment) self-compassion training. Based on the *PICOT* framework, *Participants* were healthy volunteers, randomized to four conditions: (i) the main experimental *Intervention:* tVNS plus self-compassion mental imagery training (SC-MIT) or one of three *Comparators*, comprising the remaining factorial combinations of the Stimulation and Mental Imagery Training (MIT) factors: (ii)tVNS plus Control-MIT, (iii) sham stimulation plus SC-MIT *or* (iv) sham stimulation plus Control-MIT. Primary *Outcomes* were state self-compassion, self-criticism, and heart rate variability (HRV). Exploratory and secondary outcomes included state mindfulness, trait self-compassion, and oculomotor attentional bias to compassion-expressing faces, assessed using eye-tracking.

If tVNS indeed augments SC-MIT, we expected to observe higher state self-compassion and HRV and lower state self-criticism after pairing tVNS with SC-MIT. Whether such effects would emerge rapidly (i.e. in the first session of tVNS+SC-MIT) or require multiple sessions was unclear (Hays et al., 2023; Ridgewell et al., 2021). As such, we explored the interaction between stimulation and MIT across two *timeframes*: acutely (*within* session 1) and cumulatively *across* eight sessions.

Findings of enhanced self-regulation following tVNS + SC-MIT would support notions of the ‘compassionate vagus’ (Di Bello et al., 2020; Porges, 2017) and provide a rationale for further studies of electroceutical-augmentation of contemplative psychotherapies targeting impairments in (self-)affiliative behavior. More provocatively, such findings might suggest a role for similar neurostimulation interventions in ‘virtue engineering’ (Hughes, 2023), given that contemplative practices promote valorized behaviors (e.g. generosity, compassion, fairness; Berryman, Lazar, & Hohwy, 2023), which, in turn, contribute to psychological health (Macaskill & Denovan, 2014).

## Methods

The study was preregistered (https://osf.io/4t9ha) as a single-site randomized controlled trial (NCT05441774). Ethical approval was granted by the University College London Research Ethics Committee (study reference: 0760/006; approval date: 11/05/2021). All procedures were in accordance with the Declaration of Helsinki and participants provided informed consent. Full methodological details are provided in a published protocol (Kamboj et al., 2023; see also, Supplementary Methods).

### Setting

Laboratory sessions were conducted in a nonclinical, academic (university) setting; remote sessions were conducted outside the lab (e.g. at participants’ homes).

### Participants

Healthy adults (*n* = 120) were recruited through online adverts. Eligible participants were 18–35 years old and fluent in English. Exclusion criteria included current treatment for mental health difficulties, cardiovascular, or inflammatory complaints, illicit drug use >2/week, hazardous alcohol consumption, history of severe mental, neurological, or cardiovascular disease, and history of adverse response to meditation (full eligibility in Supplement, Section 1.1).

### Design

**Figure 1.**
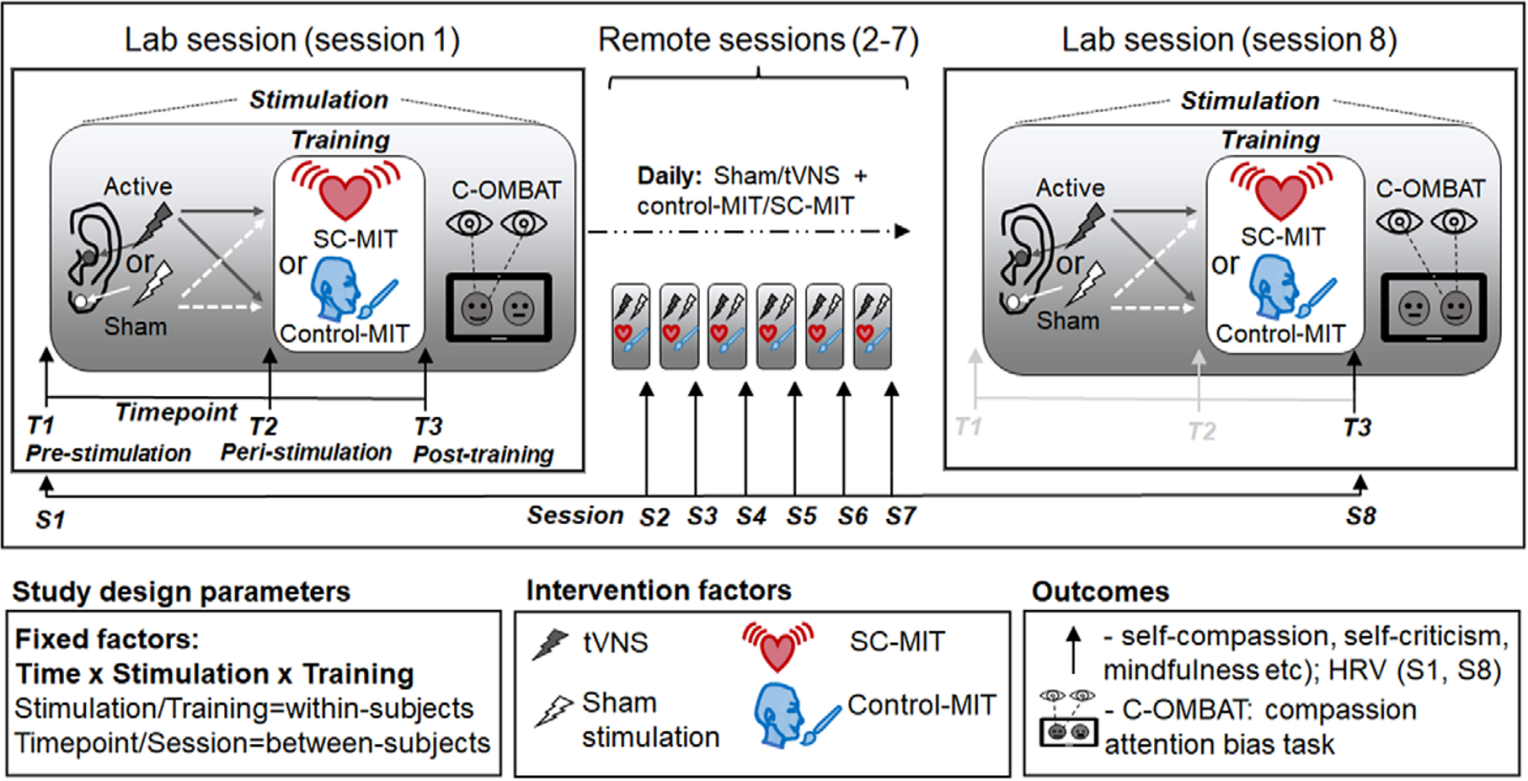
Design and procedure. The general procedure during each *lab session* (sessions 1 and 8) was identical (except for the duration of Mental Imagery Training; MIT, see text). Lab session procedures are bounded by square boxes, showing the timeline of experimental manipulations and assessment episodes (*Timepoint;* T1, T2, T3, indicated by short upward arrows). At T1, baseline assessments of HRV and state measures (self-criticism/self-compassion, mindfulness, etc.; see text) occurred *before* any stimulation/MIT. The shaded boxes within sessions 1 and −8 indicate the procedures carried out *during stimulation* on each lab session (black ‘shock’ icon on left tragus = tVNS; white ‘shock’ icon on left earlobe = sham; pulsing heart icon = SC-MIT; face/brush = Control-MIT). These procedures included the assessment of state measures and HRV at T2 following 30 min of continuous task-free stimulation. At T3, immediately after MIT, HRV and state measures were repeated, followed by the C-OMBAT. Acute (rapid) effects of stimulation and MIT were inferred from changes in state measures and HRV assessed at T1, T2, and T3 on session 1. Assessments across *Sessions* (S1–S8; long upward arrows) were intended to capture the sustained/ and cumulative effects of the *combination of* stimulation and MIT. Remote sessions (sessions 2–7) repeated the essential elements of the lab sessions: 22–24 min of task-free stimulation was followed by MIT while stimulation continued (total simulation time: 30 min). State measures were only administered *once* at the post-MIT timepoint during each remote session (corresponding to T3 in the lab session); HRV was not assessed during sessions 2–7).

A fully factorial design was used, with two treatment factors, each with two levels: stimulation (tVNS vs. sham) and mental imagery training (MIT; self-compassion-MIT vs. Control-MIT). As such, participants (balanced for sex) were randomized evenly (1:1:1:1; *n* = 30/group) to *Sham + Control-MIT*, *Sham + SC-MIT*, *tVNS + Control-MIT*, or *tVNS + SC-MIT* using a random number generator (https://www.random.org/) in blocks of *n* = 40 as outlined in Kamboj et al. (2023). Outcomes were assessed repeatedly within- and between-sessions (Figure 1). Further details on the procedure are presented in the Supplement (Section 1.4) and Kamboj et al. (2023).

### Interventions

#### tVNS and sham stimulation

tVNS devices (Parasym Ltd, United Kingdom) were used to deliver current pulses via electrodes attached to the external ear. Stimulation parameters are outlined in full in the Supplement (Table S1). Briefly, 200 μs rectangular pulses at 20 Hz were delivered at individually adjusted intensities based on participants’ sensory thresholds to the left tragus (active stimulation/tVNS) or left earlobe (sham).

There were a total of eight daily stimulation (+MIT) sessions (Figure 1). In session 1 (in-lab), after baseline measures were taken, participants received 30 min of tVNS or sham stimulation in the absence of any concurrent tasks or assessments. Stimulation concurrent with the assigned mental imagery training (SC-MIT or Control-MIT) followed by other assessments/tasks (Figure 1) continued for the remainder of the session (~77-min total stimulation on session 1). Participants repeated their assigned stimulation (tVNS/sham) remotely during sessions 2–7 (30 min/day: 22–24-min task-free followed by 6–8-min concurrently with assigned daily MIT). For the final session (session 8), participants returned to the lab and repeated essential aspects of session 1: an initial 30-min task-free stimulation period was followed by an additional ~34 min of stimulation (until the end of the session), consisting of a period of MIT-concurrent stimulation (~9 min) and stimulation concurrent with the remaining assessments (state questionnaires and C-OMBAT; ~64 min total stimulation). The total duration of stimulation across the entire trial was, therefore, ~320 min. Compliance with stimulation on sessions 2–7 (the remote sessions) was determined indirectly via webpage data (see Supplementary Results, Section 2.2.3) and self-ratings of adherence.

#### Mental imagery training

Each MIT session occurred *during* daily stimulation and was guided by audio-recorded instructions. The two standardized MITs, namely SC-MIT and Control-MIT, were designed to be equally credible and generate equivalent positive expectations regarding ‘efficacy’. SC-MIT was derived from Gilbert’s compassion-focused therapy (Gilbert, 2014). Briefly, this involved asking participants to imagine an ideal compassionate being while encouraging physical sensations associated with warmth, self-care, and so forth. Control-MIT was designed especially for this study and involved instructing participants to imagine themselves drawing/painting a specific face (presented at the start of the study) and developing and elaborating this mental image. Further detail is provided in the Supplement Section 1.2.3 (verbatim MIT scripts can be found at https://osf.io/sf295).

#### Condition concealment

Participants were unaware of the study’s aims or that there were multiple conditions to which they could be randomized. The pre-study participant information sheet only referred to tVNS, not sham, and a ‘mental imagery task’, but no further details were disclosed until session 1, prior to consent. Thus, all participants were led to believe they would receive a combination of active vagal stimulation via the external ear *plus* active mental imagery training without specifying the different imagery conditions. Researchers were not blind but had a minimal active role in data-collection (self-report data were entered directly onto the computerized survey program by participants, without verbal prompts from researchers), and intervention delivery, which was fully standardized across participants via written or audio instructions, and was largely automated.

### Measures

#### Prespecified outcomes

Primary outcomes were self-reported *state self-compassion* and *self-criticism* (assessed using the Self-Compassion-Self-Criticism scale (SCSCS; Falconer, King, & Brewin, 2015), and HRV (see *Psychophysiology* below). Secondary outcomes were *state mindfulness* (State Mindfulness Scale; SMS; Shoham et al., 2017)*, safe-warm positive affect* (subscale of Types of Positive Affect Scale, TPAS; Gilbert et al., 2008)*, general positive and negative affect* (Positive and Negative Affect Schedule [PANAS]; Thompson, 2007) and eye-tracking metrics (see below).

#### Baseline characteristics and ‘trait’ measures

Demographics and other baseline variables were assessed before T1 during session 1. Baseline distress was evaluated using the Depression Anxiety and Stress Scale (Lovibond & Lovibond, 1995). Trait self-compassion and aversion to self-compassion were assessed respectively using the Sussex Oxford Compassion Scale (SOCS-self; Gu et al., 2020) and Fear of Self-Compassion Scales (Gilbert, McEwan, Matos, & Rivis, 2011) and trait mindfulness with the Five Facet Mindfulness Questionnaire (FFMQ 15-item version; Gu et al., 2016). The SOCS-self and FFMQ-15 were repeated on session 8 allowed these to serve as additional exploratory outcomes assessing cumulative/sustained effects of stimulation+MIT.

#### State measures

‘State’ [mindfulness/self-compassion] here refers to momentary, changeable, or episodic psychological conditions, as opposed to relatively stable ‘traits’. State questionnaires (see ‘*Prespecified outcomes*’ above) administered at T1 (pre-stimulation), T2 (peri-stimulation/pre-MIT), and T3 (post-MIT, while stimulation continued) were used to assess acute (i.e. rapidly acting) effects of stimulation + MIT. The post-MIT (T3) timepoint measures were repeated daily after remote stimulation + MIT sessions (sessions 2–7; Figure 1) and the final (in-lab) session (session 8) to evaluate the cumulative/sustained effects described below.

#### Manipulation checks

To ensure that any observed effects could be attributed to tVNS and/or SC-MIT, rather than potential treatment-related confounders, a number of manipulation checks were performed.

We assessed the expectancy and credibility of participants’ assigned treatments, their post-intervention beliefs about the effects of stimulation on mental imagery abilities, treatment fidelity, stimulation ‘dose’, and (side-)effects that might contribute to (or indicate) differential placebo effects or unintentional unblinding. A ‘dummy outcome’ – the Vividness of Facial Imagery Questionnaire (VFIQ) – was included alongside the state measures (above) to assess the vividness of the ‘target face’ presented at the start of session 1. This face image was the designated stimulus used by participants in the Control-MIT condition (see Kamboj et al., 2023). By explicitly assessing its ‘efficacy’ using the VFIQ, we were aiming to increase the credibility of the Control-MIT.

#### Psychophysiology

As indicated in Figure 1, cardiac and eye-tracking measures were only collected during sessions 1 and 8 (see Kamboj et al., 2023). A lead-II ECG configuration (Bodyguard-2, Firstbeat, Finland) was used to sample interbeat intervals in 5-min epochs from seated participants. Acute HRV effects were assessed on session 1 at T1–T3, and cumulative effects between baseline (T1; session 1) and the final sampling point (T3; session 8). HRV metrics, namely the Root Mean Square of Successive Differences (RMSSD) and high-frequency power (HF-power), were derived using the Kubios software package (Tarvainen et al., 2014).

Attentional bias toward signals of interpersonal compassion was measured using eye-tracking metrics obtained during an incidental face-preference task (Compassion-Oculomotor Biased Attention Task [C-OMBAT]; Figure S2; additional details on the C-OMBAT in Section 1.3.2 of the Supplement). Participants viewed two adjacent images of the same ‘synthetic’ female face, responding with a button press to indicate which they preferred (48 trials). One face had a neutral expression, and the other (of the same ‘synthetic individual’) expressed ‘empathic-compassion’ (Supplement, Figure S2; Falconer et al., 2019). Attentional bias to compassion was inferred from longer gaze duration and larger pupil response to compassion-expressing versus neutral faces (Blini & Zorzi, 2023). C-OMBAT performance was measured twice *after* T3 assessments on sessions 1 and 8. As such, there was no ‘baseline’ assessment of attentional bias.

### Statistical analyses

Analyses were performed using Stata (v18, Stata Corp). Additional details are provided in the Supplement (Section 1.5). State self-compassion, self-criticism, and HRV were preregistered primary outcomes. Analyses of other outcomes were preregistered but are formally exploratory. With the exception of state mindfulness and eye-tracking measures, exploratory analyses are presented in the Supplementary Results.

The acute and cumulative effects of stimulation and MIT were, respectively, analyzed over two timeframes: *timepoint* (three levels: pre-stimulation, peri-stimulation, and post-MIT; T1–T3) and *session* (eight levels: sessions 1*–*8 *or* two levels: sessions 1 *and* 8, depending on frequency and timing of measurement of the outcome in question). As such, our research questions relating to the interacting effects of stimulation and MIT over time were tested in a series of omnibus three-way Time (Timepoint *or* Session) × Stimulation × MIT (generalized) linear mixed models ((G)LMMs) with per-participant intercepts as a random factor. Where significant interactions were found, these were probed using visualization and decomposition at lower levels (e.g. simple effects), followed by informative pairwise tests.

Between-group univariate tests (and the associated effect sizes; *d_(Between)_*) were used to compare tVNS+SC-MT versus the average of the other three groups acutely (T3, session 1) and cumulatively (final assessment timepoint (T3), session 8; Figure 1). Repeated measure effect sizes (between timepoints/sessions *within* a specific group: *d_(Within)_*) incorporated an adjusted standard deviation term that accounted for the correlation between repeated measures (Lenhard & Lenhard, 2022).

The sample size was based on a sensitivity analysis described in Kamboj et al. (2023), indicating *n* = 30/group would achieve a power of 0.80 (α = 0.05) to detect effects with a *d_(Between)_* ≥ 0.74 between tVNS+SC-MIT and an alternative condition. With the same α and power, *n* = 30 /group was also sufficient for *d_(Within)_* = 0.53 between two timepoints in any one of the four groups, and *n* = 60 was adequate to detect with-condition effect sizes of *d* ≥ 0.37 between timepoints within levels of a single (e.g. stimulation) factor. The presence/impact of missing data was minimal (≤3.23% missing values across all cells for the primary outcomes). No imputation methods were used.

All tests were two-tailed with α ≤ 0.05.

## Results

### Participants

Participant flow from recruitment and allocation through to study completion is outlined in the CONSORT flowchart (Supplementary Figure S1). Participant characteristics are described in Table 1 (also Supplementary Table S2).

**Table 1.** Demographics and baseline characteristics. *Mean* (*SD*) or *n* (%) for the four Stimulation × MIT conditions (*n* = 120; *n* = 30/group)

	Sham + Control-MIT	Sham + SC-MIT	tVNS + Control-MIT	tVNS + SC-MIT
Age	22.63 (4.59)	23.20 (4.16)	22.73 (3.19)	23.00 (4.36)
Male: Female (*n*)	8:22	8:22	8:22	8:22
Education (yr)	15.07 (1.82)	16.00 (1.68)	16.07 (1.55)	16.03 (1.59)
Ethnicity – white (*n*; %)	9 (30%)	7 (23%)	13 (43%)	10 (33%)
Distress (DASS–21 total)	17.33 (18.82)	13.20 (11.84)	18.07 (13.16)	18.53 (14.06)
Fear of self-compassion	17.07 (13.82)	12.27 (9.02)	15.27 (12.46)	15.97 (9.48)
Trait self-compassion (SOCS)	74.90 (12.12)	77.43 (10.96)	73.63 (8.92)	73.77 (10.78)
Trait mindfulness (FFMQ–15)	50.20 (6.62)	52.27 (6.56)	51.27 (6.09)	50.67 (6.75)
Resting HRV; HF-power (ms^2^)^¶^	743.70 (829.02)	912.96 (1057.4)	788.22 (950.75)	909.75 (1028.04)
Resting HRV; RMSSD (ms)^¶^	37.80 (19.63)	43.72 (22.86)	38.58 (22.04)	40.82 (24.07)
Meditates – Yes (*n*; %)*	3 (10%)	7 (23%)	11 (37%)	6 (20%)

*Note:* Resting HRV sampled at baseline (T1) on session 1 prior to stimulation or mental imagery training (MIT). All cells are *n* = 30 except HRV metrics for the SC-MIT + tVNS and SC-MIT + Sham stimulation groups, which each had one datapoint missing (i.e. *n* = 29) due to technical difficulties. *N = 27 participants across the four groups reported using mind–body practices: “yoga” or “Pilates” (*n* = 17); “meditation” (*n* = 6), “mindfulness” (*n* = 2), “vipassana” (*n* = 1), and “transcendental meditation” (*n* = 1). Additional measures are detailed in the Supplement (Table S2).

Abbreviations: DASS-21 (total) = Depression Anxiety and Stress Scale, the raw total was multiplied by two to correspond with the scoring of the 42-item DASS; SOCS = Sussex Oxford Compassion Scale-self version; FFMQ-15 = Five Facet Mindfulness Questionnaire; HRV=Heart Rate Variability; HF-power = High-Frequency power; RMSSD = Root Mean Square of Successive Differences.

### Manipulation checks

The results of manipulation checks are detailed in the Supplement (Tables S3–S5; Figures S3–S5). Briefly, the effects of the two MITs were selective for their respective target outcome: SC-MIT improved state self-compassion (see below); control-MIT selectively improved target-face imagery vividness. Fidelity measures suggested high levels of adherence across stimulation and MIT conditions. The four groups showed no significant differences in intervention expectancy, credibility, or perceived efficacy. Stimulation duration and amplitude (mA), and occurrence and severity of stimulation-related (side-)effects also did not differ between stimulation conditions, except for ear pain severity (higher in tVNS versus sham), although this did not covary with any outcome (see Supplement Section 2.2; Tables S3–S5; Figures S3–S5).

### Primary outcomes

#### Self-reported self-compassion

**Figure 2.**
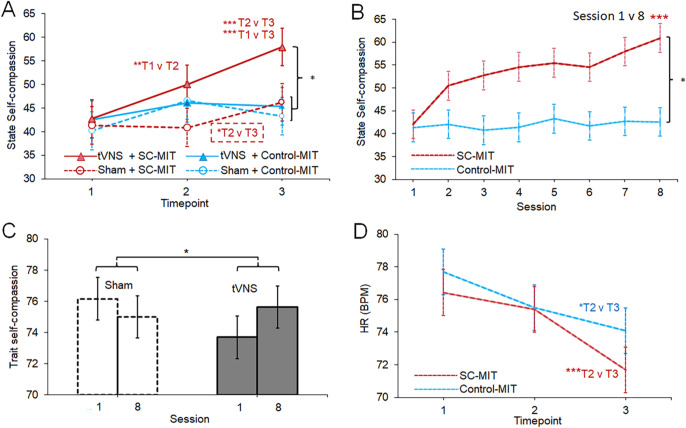
Stimulation and MIT effects on state self-compassion and heart rate on session 1 and across eight sessions. (a) Within-session acute effects of stimulation and MIT between T1 (pre-stimulation), T2 (peri-stimulation), and T3 (post-MIT) on session 1. Significant pairwise comparisons are indicated (e.g. T1 v T2). (b) Timepoint × MIT condition effects on heart rate (BPM: beats/minute) averaged across stimulation conditions. (c) Session × MIT effects on state self-compassion (SCSC self-compassion subscale). (d) Session × Stimulation effects on trait self-compassion (SOCS-S: Sussex Oxford Compassion Scale, self-version). Displayed values are estimated marginal *Means* ± *SE* Pairwise comparisons: *p < 0.05; **p < 0.005; ***p < 0.001.

The acute effects of stimulation and MIT are shown in Figure 2a and are suggestive of an increase in state self-compassion across the three timepoints on session 1 in the tVNS+SC-MIT group relative to the other conditions. This was reflected in a significant Timepoint × Stimulation × MIT interaction (*χ^2^*(2) = 7.40, *p* = 0.025, Figure 2a). Simple effects analyses confirmed significant timepoint-dependent increases in state self-compassion in the tVNS+SC-MIT (*p* < 0.001) *and* sham+SC-MIT (*p* = 0.034) groups. However, pairwise comparisons in sham+SC-MIT showed no T1→T2 change in state self-compassion (*p* > 0.99, *d_(Within)_* = −0.04), consistent with the absence of an effect of sham stimulation alone (i.e. before SC-MIT), whereas the moderate-large T2→T3 increase in self-compassion was consistent with the expected effect of SC-MIT alone (*p =* 0.019, *d_(Within)_* = 0.51). By contrast, tVNS+SC-MIT showed (i) a significant, large T1→T2 increase (*p* < 0.001; *d_(Within)_ =* 0.81), and (ii) a further significant and large T2→T3 increase (*p* = 0.001; *d_(Within)_ =* 0.84). Thus, (iii) the total effect of tVNS *plus* SC-MIT (T1→T3) was also highly significant and, by convention, very large (*p* < 0.001, *d_(Within)_* = 1.05; Figure 2a). Furthermore, univariate analysis of state self-compassion at T3 showed that state self-compassion was significantly higher in tVNS+SC-MIT compared to the average of the other conditions (*p* = 0.01, *d_(Between)_* = 0.56). Overall, these findings are consistent with an augmentation of SC-MIT by tVNS.

Considering next the cumulative/sustained effects of stimulation and MIT across sessions, we found no evidence for a three-way or two-way, Session × Stimulation interaction (*p* values ≥0.560), but a pronounced Session × MIT interaction (*χ^2^*(7) = 58.73, *p* < 0.001). This reflected a significant increase in state self-compassion with SC-MIT (averaged across stimulation; simple effects: *p* < 0.001) but not with Control-MIT (*p* = 0.899; Figure 2b). All pairwise comparisons between session 1 and each subsequent session in SC-MIT (averaged across stimulation) were highly significant (*p* ≤ 0.001) and moderate-large (*d_(Within)_* = 0.43–0.88). The SC-MIT versus Control-MIT comparison on session 8 (the most appropriate comparison for determining *sustained/cumulative* between-group differences) was also significant and moderate-large (*p* < 0.001, *d_(Between)_ =* 0.70).

To complement this analysis of sustained/cumulative effects on *state* self-compassion, we also examined the Session (two levels: sessions 1 and 8) × Stimulation × MIT effects on *trait* self-compassion (SOCS-self), an exploratory outcome. Although SOCS-self showed no interactions involving MIT (*p* ≥ 0.40), there was a significant Session × Stimulation interaction (*χ*^2^ (1) = 4.16, *p* = 0.042), reflecting an increase in trait self-compassion following eight sessions of tVNS but not sham, regardless of MIT condition (Figure 2c).

#### Self-reported self-criticism

Stimulation did not moderate the effects of MIT on state self-criticism across timepoints or sessions (two- and three-way interactions: *p* ≥ 0.273). However, there were significant Timepoint × MIT (*χ^2^*(2) = 15.16, *p* = 0.001) and Session × MIT interactions (*χ^2^*(7) = 24.43, *p* = 0.001) reflecting larger acute and cumulative/sustained reductions in state self-criticism with SC-MIT (averaged across stimulation condition; Supplementary Figure S6; Table S7). Comparing *between* MIT conditions, self-criticism was significantly lower in SC-MIT relative to Control-MIT at the end of session 1 (*p* = 0.034) and session 8 (*p* = 0.016). Self-criticism results are reported in greater detail in the Supplement (Section 2.3.2; Table S7).

#### Heart rate variability

To determine whether the acute increase in state self-compassion observed on session 1 in the tVNS+SC-MIT group (Figure 2a) was accompanied by increased cardiovagal activity, Timepoint × Stimulation × MIT effects were evaluated for RMSSD and HF-power using GLMMs. Both metrics increased with time (timepoint and session; p ≤ 0.001), but neither showed any interaction with stimulation *or* MIT (*p* ≥ 0.28; further details in the supplement; Figure S7; Table S8). Controlling for potential confounders or inclusion of potential moderators in the GLMMs did not affect these results (Supplement, Section 2.3.3). Exploratory Bayesian analysis provided moderate evidence for the null (BF_01_ ≥ 3.32; Supplement Section 2.3.3.2, cf. Wolf et al., 2021). By contrast, simple *heart rate* was a sensitive differential indicator of acute effects of MIT condition (Timepoint × MIT: *χ^2^*(2) = 6.45, *p* = 0.040; Figure 2d), but not stimulation (*p* ≥ 0.492). Although both MIT conditions showed significant reductions in HR across timepoint (simple effects: *ps* < 0.001), the T2→T3 reduction was substantially larger in SC-MIT (*p* < 0.001, *d =* −0.93), than in Control-MIT (*p* = 0.019, *d* = −0.27). On the other hand, the Control-MIT versus SC-MIT difference at T3 was negligible (*d_(Between)_ =* 0.17) and nonsignificant (*p* = 0.352).

### Exploratory outcomes

#### Self-reported mindfulness

**Figure 3.**
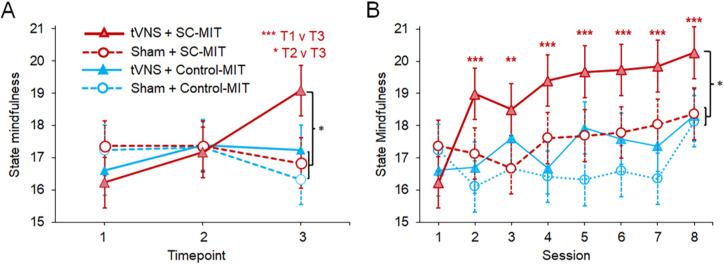
Stimulation and MIT effects on state mindfulness on session 1 and across eight sessions. (a) State mindfulness (five-item SMS; Shoham et al., 2017) at T1, T2, and T3 in each combination of levels of stimulation and MIT on day 1; (b) State mindfulness across days. Values are estimated marginal *Means* ± *SE.* Pairwise comparisons: *p < 0.05, **p < 0.01, ***p < 0.001.

State mindfulness (Figure 3a) showed a pattern of acute effects that partially resembling those seen with state self-compassion (cf. Figure 2a). This was represented by a Timepoint × Stimulation (χ^2^(2) = 10.37, *p* = 0.006) but not a Timepoint × MIT (χ^2^(2) = 3.67, *p* = 0.160) or Timepoint × Stimulation × MIT interaction (χ^2^(2) = 1.70, *p* = 0.428). Simple effects analyses confirmed a significant timepoint effect only with tVNS (averaged across MIT: *p* = 0.006), but not sham stimulation (*p* = 0.318). Pairwise tests of tVNS (averaged across tVNS+SC-MIT and tVNS+Control-MIT) showed that although sequential timepoints did not differ in either stimulation condition (*p* ≥ 0.316), the *overall* T1→T3 increase in state mindfulness was only significant in the tVNS conditions (tVNS+Control-MIT and tVNS+SC-MIT averaged, *p* = 0.004; *d* = 0.41).

As shown in Figure 3a, however, tVNS’s effects appeared to be driven by its combination with SC-MIT, especially between T2 and T3. The significant pairwise T1→T3 comparison in the tVNS conditions mentioned above is, therefore, diluted through averaging of tVNS+SC-MIT and tVNS+Control-MIT. To disentangle the additional specific effects of SC-MIT versus Control-MIT, we performed separate simple effects analyses in tVNS+SC-MIT and tVNS+Control-MIT. This showed no significant change in state mindfulness across timepoints in tVNS+Control-MIT (*p* = 0.549), whereas tVNS+SC-MIT showed a clear increase across timepoints (*p* < 0.001). Pairwise comparisons in tVNS+SC-MIT showed significant T2→T3 (*p* = 0.030, *d_(Within)_ =* 0.51) and T1→T3 (*p* < 0.001*, d_(Within)_* = 0.68) increases. Moreover, state mindfulness levels at T3 in tVNS+SC-MIT were significantly higher versus the average of the other groups (*p* = 0.02, *d_(Between)_* = 0.50; Figure 3a).

Cumulative/sustained changes in state mindfulness across the eight sessions are shown in Figure 3b. As seen acutely (Figure 3a), Figure 3b appears to show a differential enhancement of state mindfulness with tVNS+SC-MIT. However, rather than an overall three-way (Session × Stimulation × MIT) interaction (χ^2^(7) = 3.30, p = 0.856), this effect was reflected in Session × Stimulation (*χ^2^*(7) = 18.08, *p* = 0.012) and Session × MIT: (*χ^2^*(7) = 15.84, *p* = 0.027) interactions. Simple effects analyses performed at each level of each factor showed significant increases in state mindfulness in tVNS (*χ^2^*(7) = 40.97, *p* < 0.001) but not sham (*χ^2^*(7) = 12.93, *p* = 0.074) and in SC-MIT *χ^2^*(7) = 34.35, *p* < 0.001) but not Control-MIT (*χ^2^*(7) = 10.42, *p* = 0.166). This pattern of simple effects suggested that increases in state mindfulness were indeed specific to tVNS+SC-MIT. Pairwise comparisons of state mindfulness levels in tVNS+SC-MIT between session 1 and subsequent sessions were all significant (*p* ≤ 0.001; Figure 3b), reflecting steady increases in effect size across sessions (e.g. session 1 vs. session 8: *p* < 0.001; *d_(Within)_* = 0.54). Consistent with a specific increase in state mindfulness in the tVNS+SC-MIT group across sessions, state mindfulness was significantly higher in tVNS+SC-MIT compared to the average of the other conditions at the final assessment timepoint on session 8 (*p* = 0.039, *d_(Between)_ =* 0.44; Figure 3b). By contrast, *trait* mindfulness (FFMQ-15) scores did not change appreciably between sessions 1 and 8 (*χ^2^*(1) = 0.16, *p* = 0.686), with no two- or three-way interactions (*p* ≥ 0.241).

### Other state measures

The effects of stimulation and MIT conditions described above appeared to be contemplative outcomes. In particular, acutely, there were no stimulation or MIT condition effects on PANAS (positive or negative) or TPAS-safe-warm (*p* ≥ 0.135). Similarly, there were no differential cumulative effects of stimulation or MIT on PANAS-negative or TPAS-safe-warm (*p* ≥ 0.277), although a Session × MIT effect on PANAS-positive (*p* = 0.033) appeared to reflect instability in positive affect across sessions rather than a consistent effect of either training condition (Supplement, Section 2.3.4; Figure S8).

### Oculomotor attentional bias to compassionate faces

**Figure 4.**
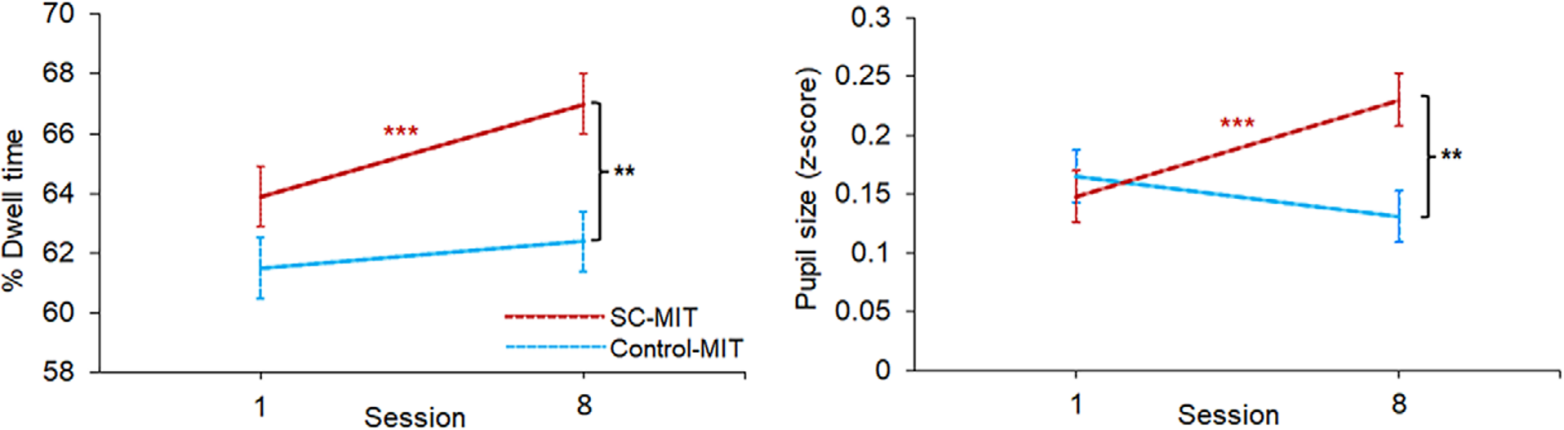
Oculomotor attentional bias on faces expressing compassion. Between-session change and between-group differences in attentional bias to compassion faces in the two MIT conditions (collapsed across stimulation conditions), expressed as (a) % gaze duration (dwell-time) on compassion faces versus total gaze duration (Dwell (compassion)/(Dwell (compassion) + Dwell (neutral)) and (b) pupil size in Z score units. Values are estimated marginal *Means* ± *SE.* Pairwise comparisons. Pairwise tests: ***p < 0.001; **p < 0.005.

Attentional bias to interpersonal signals of compassion was assessed using the C-OMBAT. Dwell-times on compassion-expressing faces appeared to increase from session 1 to 8 in response to SC-MIT (averaged across stimulation condition; Session × MIT interaction; *χ^2^*(1) = 4.83, p = 0.028; Figure 4a). Pairwise tests showed that dwell-time bias was significantly higher in SC-MIT relative to Control-MIT only on session 8 (*p* = 0.0026, *d_(between)_* = 0.61; cf. session 1: *p* = 0.193, *d_(between)_* = 0.31). In addition, the interaction partly reflected a significant session 1→8 increase only in the SC-MIT condition (*p* < 0.001, *d_(Within)_* = 0.57; cf. Control-MIT: *p* = 0.369, *d_(Within)_* = 0.17). There were no session-dependent interactions involving stimulation (*p* ≥ 0.100).

Pupil size was also differentially responsive to MIT conditions across sessions (Session × MIT: χ^2^(1) = 11.88, *p* < 0.001; Figure 4b). The interaction reflected a significant session 1→8 increase in pupil size to compassionate faces only in SC-MIT (averaged over stimulation condition: *p =* 0.003, *d_(Within)_* = 0.46, cf. Control-MIT: *p* = 0.271, *d_(Within)_* = −0.186). In addition, pairwise comparisons showed significantly larger pupil size in the SC-MIT condition relative to Control-MIT only on session 8 (*p* = 0.003, *d_(Between)_* = 0.60; session 1: *p* > 0.99, *d_(Between)_* = −0.10). Again, there was no evidence of an effect of stimulation condition across sessions (interaction effects; *p ≥* 0.093).

To explore the possibility that differential effects of stimulation on eye-tracking metrics might be determined by the compassion-related variable that showed Stimulation × Session effects – trait self-compassion – we examined the association between ∆pupil size (session 1→8) and ∆SOCS-self (session 1→8) in the two stimulation conditions (Figure S9). This showed a significant positive correlation in the tVNS (*r*(56) = 0.35, *p* = 0.007), but not sham condition (*r*(55) = −0.05, *p* = 0.696). The strength of these associations differed significantly (*z* = 2.18, *p* = 0.029).

## Discussion

The interactions between stimulation and MIT outlined here support the idea that complex, explicit forms of cognitive-affective self-regulation (state and trait self-compassion; state mindfulness) can be modulated through *peripheral* rather than central brain circuit-level neurostimulation (cf. Badran et al., 2017; Di Bello et al., 2023). They also support theoretical and empirical claims that the vagus nerve has a central role in compassionate responding (Di Bello et al., 2020; Kirby, Doty, Petrocchi, & Gilbert, 2017; Petrocchi, Di Bello, Cheli, & Ottaviani, 2022; Petrocchi & Ottaviani, 2024; Sherwell & Kirby, 2024; Stellar & Keltner, 2017). Specifically, our main finding of a Timepoint × Stimulation × MIT interaction for state self-compassion on session 1 reflected a larger effect of tVNS+SC-MIT relative to the other conditions. Indeed, the 15-point (T1→T3) increase in state self-compassion is comparable to the SC-MIT-augmenting effect of the powerful entactogen, 3,4-methylenedioxymethamphetamine (Kamboj et al., 2015). However, the differential effects of tVNS+SC-MIT on *state* self-compassion were not sustained across sessions, possibly because the large effects of SC-MIT alone obscured the subtler additional effects of tVNS across sessions. On the other hand, *trait* self-compassion increased between sessions 1 and 8 after repeated tVNS but not sham stimulation. The latter effect was not moderated by the MIT condition. These findings might suggest differential sensitivity of distinct self-compassion outcome measures to rapid and sustained/cumulative effects of tVNS.

The acute and sustained/cumulative effects of tVNS+SC-MIT on state mindfulness – although exploratory – were also noteworthy, especially given that we deliberately omitted any specific acceptance, nonjudgment, or focused attention instructions from the SC-MIT training (see https://osf.io/sf295). Although these effects were represented by two-way (Timepoint /Session × Stimulation) rather than three-way interactions, the descriptive pattern, supported by simple and pairwise effects, suggested an augmentation of SC-MIT by tVNS. These findings are consistent with collinearity between measures of mindfulness and self-compassion (Ferrari et al., 2019; Miller & Verhaeghen, 2022; Tirch, 2010) and the observation that interventions designed to enhance one of these self-regulatory capabilities also enhance the other (Jazaieri et al., 2014; Kuyken et al., 2010). Our findings complement this literature by showing cross-sensitivity between contemplative states in their response to augmenting biological strategies.

Our findings also suggested that oculomotor attentional metrics have utility as indices of responsivity to behavioral self-compassion interventions. Although tVNS did not directly affect these measures, the differential effect of tVNS versus sham on the relationship between ∆SOCS-self and ∆pupil-size suggests a common underlying mechanism activated by tVNS affecting both nonverbal *and* explicit expressions of self-compassion. By contrast, increases in self-compassion and mindfulness were not preceded or accompanied by differentially higher HRV in the tVNS and/or SC-MIT conditions. The lack of effect of SC-MIT is inconsistent with well-powered studies that showed increased HRV following compassion-induction procedures (Kirschner et al., 2019; Stellar, Cohen, Oveis, & Keltner, 2015). Contrastingly, the observed reduction in *heart rate* following SC-MIT does align with this prior research (Kirschner et al., 2019), although it does not necessarily imply increased vagal activity since bradycardia is also driven by sympathetic withdrawal, which has also previously been observed following behavioral self-compassion interventions (Arch et al., 2014).

The observed increases in HRV over timepoints and sessions across all conditions (Supplement) might suggest that Control-MIT (unintentionally) produced sufficiently similar (T2→T3) increases in parasympathetic activity to SC-MIT as to make the two MIT conditions indistinguishable (at least in terms of their physiological effects). This points to a general challenge in contemplative science of designing well-matched comparators that control for nonspecific characteristics of the active contemplative intervention, while lacking efficacy on the targeted processes/outcomes (e.g. parasympathetic activity, state self-compassion). Given this challenge, future studies might benefit from including passive (no-MIT) controls that allow nonspecific effects of time to be distinguished from general MIT-induced changes in HRV.

Regarding the null effect of tVNS on HRV, our results resemble other studies showing clear subjective or behavioral changes *without* accompanying changes in HRV following tVNS (Borges et al., 2020; De Smet et al., 2021; De Smet et al., 2023). These findings contribute to growing evidence for an *absence* of HRV changes following auricular-tVNS (BF_01_ ~ 25; Wolf et al., 2021). Consequently, there is a lack of reliable and convenient positive controls/biomarkers (such as HRV) for vagal stimulation (Burger, D’Agostini, Verkuil, & Van Diest, 2020), which creates interpretational challenges by extending the range of alternative explanations for changes in behavioral and self-report measures following tVNS. While we cannot definitively rule out explanations such as self-report biases, stronger expectancy, or placebo effects in the tVNS (± SC-MIT) conditions, we believe the various controls we employed make such explanations unlikely. First, the four Stimulation × MIT conditions showed no difference in expectancy, credibility, or post-intervention beliefs about the efficacy of the intervention. Second, adherence to stimulation and MIT – and hence, ‘dose’ – did not differ between conditions. Third, the intensity/occurrence of general and adverse effects was similar in the two stimulation conditions, suggesting no differential placebo effects. Fourth, the effects of tVNS+SC-MIT were specific to contemplative outcomes rather than secondary to undifferentiated changes in general affective state (e.g. Ferstl et al., 2022). However, we cannot rule out alternative *physiological* explanations for the observed psychological effects of stimulation. For example, tVNS’s effects on self-compassion and mindfulness might have reflected sympathoinhibition to a greater extent than vagal activation (Mahadi, Lall, Deuchars, & Deuchars, 2019).

## Limitations

The absence of a differential physiological indicator of vagal activation and lack of a measure of sympathetic activity represent limitations of the current study. In the case of vagal indicators, we did not prioritize the measurement of central biomarkers of tVNS-induced changes in brain activity. Examples of scalable biomarkers include pupillometric responses to *phasic* tVNS (Pervaz et al., 2024) and attenuated alpha oscillations in EEG (Sharon, Fahoum, & Nir, 2021). Although we used eye-tracking in the current study, we did not evaluate the effects of task-independent phasic vagal stimulation, which appears to be required for inducing increases in pupil size. Future studies might consider incorporating these metrics as positive controls.

Additionally, our use of SC-MIT only partially simulated clinical compassion-focused therapy practices. In particular, SC-MIT in the current study encouraged self-compassionate responding in the absence of (engagement with) overt suffering. We also did not assess MIT-related adverse effects. Finally, given the discovery-oriented/exploratory nature of this research, we examined a relatively large number of outcomes, increasing the risk of Type-I errors. As such, our findings should be considered a promising preliminary foundation for future confirmatory experimental research in which a more limited number of primary outcomes (informed by the current study) could be prespecified.

## Conclusion

Our study partially supports the notion that the vagus nerve has a role in compassionate behavior. It also suggests the potential application of tVNS in augmenting contemplative practices/psychotherapies. Further confirmatory research is essential to determine whether such neuroenhancement strategies have value as clinical interventions or as ethical modes of ‘virtue engineering’.

## Supporting information

Kamboj et al. supplementary material Kamboj et al. supplementary material

## Supplementary material

The supplementary material for this article can be found at http://doi.org/10.1017/S0033291725101013.
