# Supplementary material for: Electroceutical enhancement of self-compassion training using transcutaneous vagus nerve stimulation: results from a preregistered fully factorial randomized controlled trial
Source: Psychol Med. 2025 Aug 4;55:e223. doi: 10.1017/S0033291725101013 (PMC12341030; doi:10.1017/S0033291725101013)
Supplement: Kamboj et al. supplementary material [file S0033291725101013sup001.pdf]

SUPPLEMENT

1. SUPPLEMENTARY METHODS

1.1. Participants

Participants were community-dwelling adults, recruited through online adverts. Potential participants were excluded if they were currently taking any medication for a psychiatric, cardiovascular or inflammatory condition, used recreational drugs >2/week or if their regular weekly alcohol consumption was considered (according to UK guidelines) to be hazardous, that is, it exceeded the equivalent of 140 ml of pure ethanol/week. Additional exclusion criteria were: receiving current mental health treatment, a history of serious mental health problems (e.g. schizophrenia), a history of cardiovascular or neurological disease, facial or ear pain or skin irritation/broken skin at the stimulation sites, pregnancy, a previous adverse response to meditation, and scores of >4 on the two, two-item mental health screeners: Patient Health Questionnaire-2 or Generalized Anxiety Disorder-2 rating scales (Kroenke et al., 2007). The flow of participants from screening through follow-up (session-8) is shown in the CONSORT flow-chart (Fig. S1). One participant (randomized to sham + SC-MIT but with no knowledge of their assignment, see below) withdrew due to illness before completing any active study procedure. They were replaced.
Of n=120 participants who started the study, 100% were retained until the end of the study (session-8) . Although perfect retention is rare in clinical trials, early-phase trials - such as the current one – can have relatively short follow-up periods and are less likely to be affected by morbidity-related or other clinical factors that contribute to dropouts.

1.2. Interventions

1.2.1 Treatment Concealment and Bias Minimization
Details on single-blinding of participants are provided in Kamboj et al (2023). Briefly, to minimize expectancy effects and conceal the true nature of the study, no reference was made in the study advert or participant information sheet to the true purpose of the study, its hypotheses or the design. In particular, participants were not aware that they would be randomized to one of four conditions (they were not aware of the multiple conditions, i.e. there was no reference in participant-facing materials to control or active stimulation or mental imagery training conditions).

The nature of the mental imagery training (MIT) tasks was concealed until session-1, and only disclosed before obtaining consent. Prior to session-1, there was also no mention of "meditation" except for a screening question enquiring about previous adverse experiences of meditation (an exclusion criterion). On session-1, MIT conditions were described as either a "self-compassion imagery exercise" or a "face-processing exercise" depending on participants' random allocation.

Researcher influences were minimized, and data integrity was protected by largely automating the procedure. Specifically, instructions were provided as on-screen directions and researcher-participant interactions during the lab visits (sessions 1 and 8) were limited and largely scripted. During these lab sessions, responses to question(naire)s were entered directly onto the computer survey program by the participant and therefore concealed from the researcher. The researchers involved in direct participant interactions therefore had no role in recording participants' responses. They were also not involved in data analysis. Conversely, the data analyst had no role in data collection.

Figure S1: CONSORT Flow chart. SC-MIT=Self-compassion Mental Imagery Training; Control-MIT=Control Mental Imagery Training

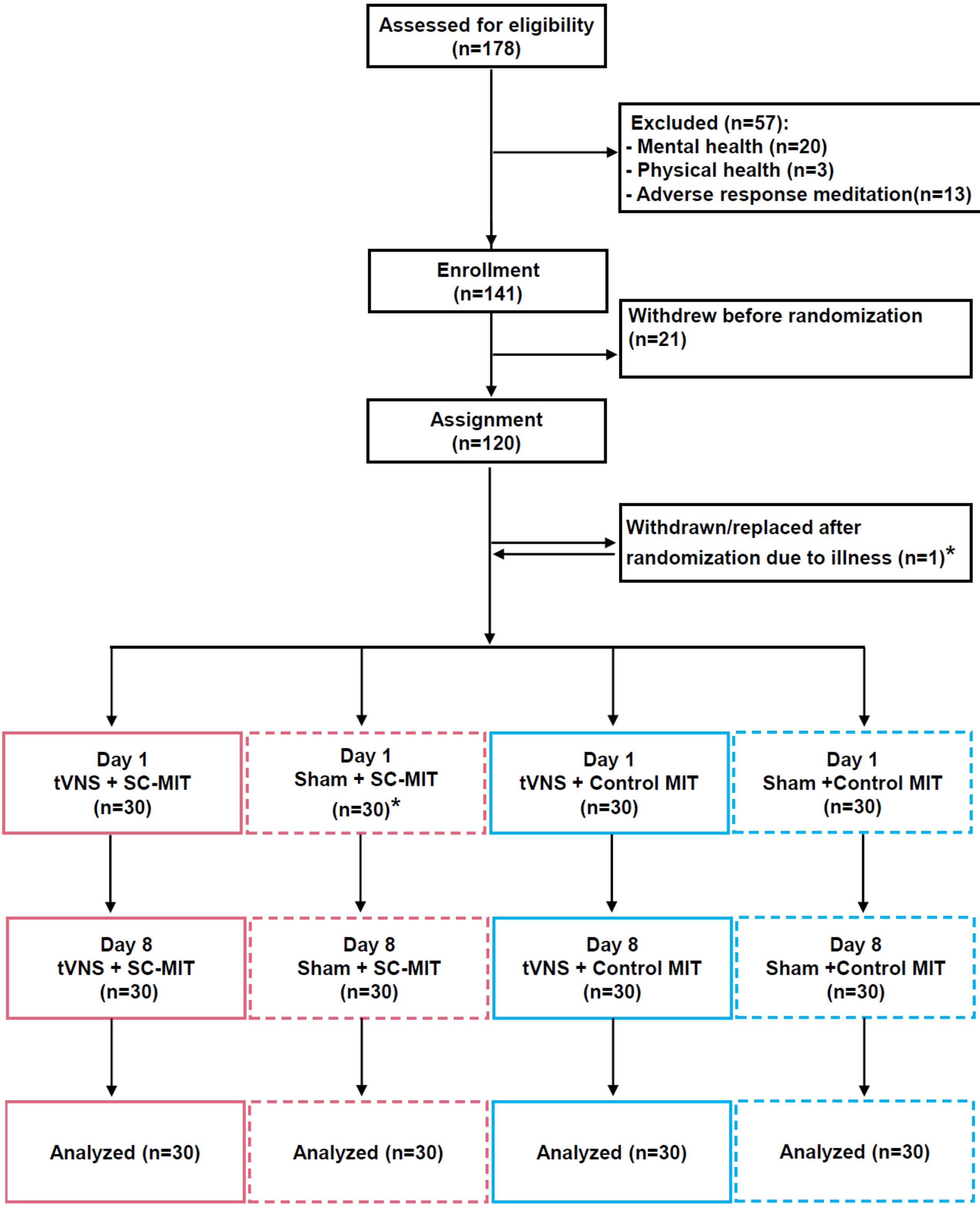

* Withdrawn/replaced after randomization to SC-MIT + sham

1.2.2. Transcutaneous vagus nerve stimulation: stimulation parameters

Stimulation parameters and device details are outlined in Table S1 according to recommended reporting standards for tVNS studies (Farmer et al., 2021). Intensity of stimulation was set individually at a suprathreshold level, at which participants experienced a clear tingling/pulsing sensation, but below the level at which stimulation was painful. The same thresholding procedure was used for stimulating the tragus (tVNS) and earlobe (sham) and was repeated on each of the eight sessions and the stimulation intensity was recorded on a 1-40 scale (each unit corresponding to 0.8 mA; see Kamboj et al, 2023).

Table S1: Stimulation parameters for tVNS

Stimulation parameter	Details
Device manufacturer	Parasym Ltd
Control	Variable current (constant voltage in tissue)
Individualization	Individualized intensity, above sensory/below pain threshold.
Direction	Preferentially afferent [Δ]
Location	Sham=Earlobe; active tVNS=tragus (anterior and posterior surface)*
Electrodes	Two, gold-plated attached via ear-clip
Duty cycle	Constant
Frequency	20 Hz
Pulse shape	Rectangular
Pulse width	200 µS
Amplitude	Individually tailored from 1-40 (each increment=0.8 mA[⁋]) [⁋⁋]
Average stimulation periods	
• Session-1(1st lab session)	77 min [ΔΔ]
• Sessions 2-7	30 min/day
• Session-8 (2nd lab session)	64 min**
• Total	≃ 321 min[⁋⁋⁋]
Timing relative to task	Task-free and task-concurrent

[Δ] Information provided by manufacturer; *Left ear; [⁋]Assumes fixed resistance of 500 Ω; [⁋⁋] Average intensities across sessions are summarized in Fig. S3. [ΔΔ]30 min task-free + plus ~47 min concurrent (until end of C-OMBAT) stimulation; **30 min task-free plus ~34 min concurrent (until end of C-OMBAT) *c.f.* published protocol (Kamboj et al, 2023). [⁋⁋⁋]Total stimulation over the eight sessions, assuming 100% adherence.

On the first lab session (session-1) participants received an initial 30-min of tVNS or sham stimulation in the absence of concurrent MIT (task-free stimulation). During this time they watched an emotionally-neutral nature documentary (Kamboj et al., 2023). The effects of this 30-min task-free[1] stimulation period were then recorded using self-report state measures and HRV (T2; see Fig. 1 in the main paper). Stimulation then continued for the remainder of the session, during which participants practiced their assigned MIT, repeated the state measures and completed the C-OMBAT (see below). The total duration of stimulation on session-1 was 77-min. A virtually identical procedure was followed on the second lab session (session-8), although the MIT practice (and hence,

[1] 'Task-free' and 'concurrent' stimulation, referring respectively to the absence or presence of a concurrent task (training), are often referred to as 'off-line' and 'on-line' stimulation respectively. Given that much of our procedure occurred remotely and responses were recorded on the study website (i.e. an alternative use of the term 'on-line'), to avoid confusion, we have deliberately avoided the 'on-line'/'off-line' terminology to refer to stimulation.

the period of concurrent stimulation) was briefer, such that the total stimulation period (including the initial 30-min task-free) was ~64-min[2].

Between the two lab sessions, i.e. sessions 2-7, participants self-administered daily sham stimulation or active tVNS at home at approximately the same time every day for 30-min. The at-home stimulation consisted of an initial 22- or 24-min of offline stimulation, followed by an additional 8-min or 6-min of concurrent stimulation while participants practiced SC-MIT or control Mental Imagery Training (Control-MIT) respectively (using abbreviated audio-records similar to those used in session-1). An on-screen timer provided a countdown for the stimulation period. Based on these daily stimulations, the (intended) total stimulation duration was $\sum_{i=1}^{8} \text{Session}_i \simeq 321$-min (assuming complete adherence).

1.2.3. Self-compassion Mental Imagery Training (SC-MIT) and Control Mental Imagery Training (Control-MIT) [3]

On session-1, the SC-MIT or Control-MIT ('draw-a-face'[4]) audio instructions were presented *during stimulation*. The scripts are available on the Open Science Framework and illustrate the parallels in the conditions in terms of number of words (duration) and general structure. The instructions were carefully designed[5] to be equally credible and to generate similar positive expectancies in the two conditions. In addition, other key attributes of the two MIT conditions were designed to be as similar as possible (Kamboj et al, 2023 for details):

Number of words: SC-MIT = 1321 words; Control-MIT = 1356 words
Complexity of language (Flesch-Kincaid level): SC-MIT = 10.5; Control-MIT = 8.4
Length of the audio recording: see below.

The instructions were presented in three sections: (i) an introductory rationale/explainer, (ii) orientation/preparation and (iii) MIT practice. In the introductory audio (section (i)), participants were told that tVNS can improve mental imagery by activating brain processes involved in either "*producing feelings of safeness and comfort, which are a prerequisite for self-compassion*" (SC-MIT) *or* "*forming and manipulating mental images*" (Control-MIT). As such, only one of these explanations was grounded in prior research and was part of the main hypotheses of the study (i.e. the vagal basis for compassionate responding in SC-MIT e.g. (Petrocchi et al., 2022) (Porges, 2017) (Stellar & Keltner, 2017)). Additionally, the rationales were designed to generate similar positive expectancies relating to the applied or clinical uses of methods for "*stimulating the vagus nerve*". Specifically, we explained that the results of the study could be used to develop "[new therapies] *for people with depression and other psychological disorders, in which self-compassion is often lacking*"

---

[2] The 77-min and 64-min durations differ slightly from the published protocol (Kamboj et al., 2023), which contained estimates rather than empirically derived durations.

[3] Design and validation of these trainings is described in detail in Kamboj et al (2023).

[4] The 'target face' was presented to all participants at the beginning of session-1 as a part of an ostensible face-rating (friendliness, approachability, trustworthiness) task. The target face was intended to provide a standardized stimulus for the Control-MIT (draw-a-face) training, the vividness of which was rated on the Vividness of Facial Imagery Questionnaire.

[5] In line with standards for transparent reporting in contemplative science regarding the qualifications and experience of the 'instructor', the interventions were designed/adapted by author SKK, a Research Clinical Psychologist with a master's degree in Mindfulness Studies and extensive experience in contemplative practice and research. SC-MIT was adapted from scripts commonly used in Compassion-Focused Therapy (CFT). Author PG is the originator of CFT and has written extensively on this modality, including treatment manuals.

(SC-MIT) or "[methods] *for improving memory for faces that would otherwise be difficult to remember… [for] application in forensic cases, for example*" (Control-MIT, i.e. 'draw-a-face training')[6].

Although instructions for the MIT conditions were carefully validated prior to use in the current study (Kamboj et al., 2023), it was important to determine whether participants actually experienced the rationales for each MIT condition to be credible and to generated positive expectancies. Therefore, credibility and expectancy were assessed after audio section (i) (the rationale/explainer; see *Manipulation checks* below).

Sections (ii) and (iii) of the audio instructions of SC-MIT were derived from Gilbert's Compassion Focused Therapy (Gilbert, 2014). However, because we wanted to avoid parasympathetic effects attributable to paced breathing (which is typically incorporated into CFT-based imagery exercises) and were interested primarily in the interaction between tVNS and *compassion* training, the SC-MIT condition deliberately excluded paced breathing instructions and avoided explicit instructions that encouraged mindful-like states. For Control-MIT, audio sections (ii) and (iii) involved instructing participants to imagine using a drawing/painting implement of their choice to recreate (paint/draw) in imagination an unfamiliar (AI-derived; see Kamboj et al, 2023) face presented as a photographic image at the beginning of session-1 (see below).

The total durations of audio recordings (i.e. sections i to iii) on session-1 were 15 and 16 min for Control-MIT and SC-MIT conditions respectively. On sessions 2-8, audio sections (i) and (ii) were superfluous and therefore omitted from the guided practices. Instead, the audio instructions during MIT practice on these sessions consisted only of abbreviated versions of audio section (iii) for each MIT condition. On sessions 2-7 (the remote sessions), participants were sent daily reminders to complete their session by logging onto the study website and following instructions on stimulation (including the thresholding procedure) and MIT practice.

1.3. Measures

1.3.1 Manipulation Checks

1.3.1.1. Credibility, Expectancy and Post-intervention Efficacy Ratings

Credibility and expectancy were evaluated using Likert scales (0=not at all; 8=very) assessing participants' appraisals of '*logic*' and '*likely success*' and if they would '*consider using*' tVNS to improve mental imagery abilities, adapting Devilly and Borkovec's (2000) expectancy and credibility questionnaire. As noted above, these ratings were obtained on session-1 after the rationale (audio section (i)). On session-8, after the final outcome measures were obtained, complementary posterior ratings of participants' beliefs about the perceived efficacy of stimulation on improving their mental imagery ability were obtained[7].

---

[6] The rationale provided to participants about the interaction between stimulation of the vagus and mental imagery training made no reference to the site of stimulation (stimulation was simply referred to as 'vagus nerve stimulation' regardless of stimulation condition). As such, all participants were led to believe they were receiving the active, augmenting form of stimulation (tVNS).

[7] The credibility, expectancy and posterior belief questions are presented in the footnote to Table S3.

1.3.1.2. Treatment fidelity: Adherence

Participants provided ratings of how closely they followed the audio instructions for the Control-MIT and SC-MIT conditions at the end of each session ("*As honestly as you can, please rate how closely you followed the audio imagery instructions*" on a 0-8 scale; see Fig. S4). In addition, measures of adherence to at-home stimulation ("*Number of days (out of 6) you performed the stimulation*") and MIT practice ("*Number of days (out of 6) you listened to the imagery instructions*") were obtained at the end of session-8. To encourage honest reporting on adherence, participants received the following instruction prior to the ratings: *"Although we encouraged you to complete the at-home tasks every day, we do understand that this can be difficult for people, and that it's sometimes impossible to do these tasks every day, or even on most days. Now that you've completed the study, it's very important for us to know how many days you actually completed the stimulation and how many days you followed the imagery instructions. We can then factor this into our statistical analysis and get a more accurate estimate of the real effects of stimulation. Please answer as honestly as possible. Your responses will have no effect on your payment for participation!*".

These self-reported measures of adherence were supplemented with data from participants' website-use, namely the frequency and duration of their interactions with the stimulation and MIT instruction/countdown pages on the online study portal.

1.3.1.3. Treatment Fidelity: Selectivity of Mental Imagery Training Effects

Treatments delivered with a high degree of fidelity are likely to be selective (i.e. selectively affect the targeted process/outcome). In our case, we expected that SC-MIT would selectively increase self-compassion and decrease self-criticism whereas Control-MIT would show a target-specific increase in facial mental imagery performance (i.e. ratings of vividness of the target face).

The effects of SC-MIT ± tVNS are outlined extensively in the main paper and clearly showed that only those in the SC-MIT condition showed a reliable increase in the 'target-specific' outcome for SC-MIT, i.e. self-reported state self-compassion. To determine if the effects of the Control-MIT were similarly selective (i.e. had selective effects on the target-specific outcome, namely target-face mental imagery vividness) we used the Vividness of Facial Imagery Questionnaire (VFIQ). The VFIQ was primarily designed as a dummy outcome to increase the credibility of - and generate positive expectancies about - Control-MIT (see Kamboj et al, 2023). It consisted of three items assessing vividness on a 7-point rating scale of mental imagery for (i) a relative/friend, (ii) best friend from primary school, and (iii) the 'target face', namely the AI-generated photographic image presented at the start of the study. Only ratings from the latter item ("*If I imagine the face of the person that I rated for friendliness, trustworthiness and approachability at the beginning of the lab session, I have a perfectly clear and bright image*"; 1=*not at all true*; 7=*very true*) are reported below. Despite the VFIQ being devised as a dummy outcome, ratings from the latter item were informative as a manipulation check to determine if the Control-MIT indeed selectively improved facial mental imagery vividness in Control-MIT but not the SC-MIT condition.

1.3.1.4. General and Adverse Effects of Stimulation

Ratings (0=not at all; 8=severe) of general stimulation effects (tingling, pulsing, discomfort) and adverse effects across the preceding week were obtained at the end of session-8. Except for ear pain, the incidence of adverse effects was low. As such - with the exception of ear pain - we only report the incidence (any severity>0) of adverse effects of stimulation.

1.3.1.5. General Effects of Mental Imagery Training: Mental Imagery Characteristics
Participants rated the characteristics of their mental imagery (as experienced during the MIT period) at the end of each session using a three-item nine-point rating scale. Image *clarity* and *ease* (of formation) of imagery were rated on a 0-8 scale (0=not at all; 8=extremely) and *valence* on a -4 (very negative) to +4(very positive) scale.

1.3.2. Additional Details on the Compassion-Oculomotor Biased Attention Task (C-OMBAT)
Participants were presented with two horizontally adjacent synthetic face images[8] of the same individual on a 21-inch, 1280 x 1084 resolution monitor. They were told that we were interested in determining which face they preferred. One of the pair of faces had a neutral expression, and the other was the same individual expressing compassion (Falconer et al., 2019). Face pairs were presented for 3000ms, during which time, participants only had to look at the faces (no other response was required). After the 3000ms stimulus presentation period, they were prompted to indicate by button-press which face they preferred (Fig. S2). After responding (no time limit), a fixation cross appeared for >500ms followed by the next trial.

During the viewing period participants' eye movements and pupil size were monitored via an eyetracking camera placed approximately 60 cm from their eyes (Eyelink 1000, SR Research, Canada). Head movements were minimised by placing participants' heads in a chin/forehead rest during the task.

The compassion faces could vary in intensity (25, 50, 75 and 100%) and appeared pseudo-randomly on the left or right of the screen. Here we report on the 48 trials of neutral (0% compassion) versus 100% compassion faces (see Kamboj et al, 2023).[9] Attentional bias (towards compassionate faces) was defined as larger pupil size or longer proportionate gaze duration on 100% compassionate faces (e.g. $dwell_{(100\%\ compassion)}/(dwell_{(100\%\ compassion)}+dwell_{(neutral)})$).

Figure S2. Compassion-Oculomotor Biased Attention Task (C-OMBAT): task structure.

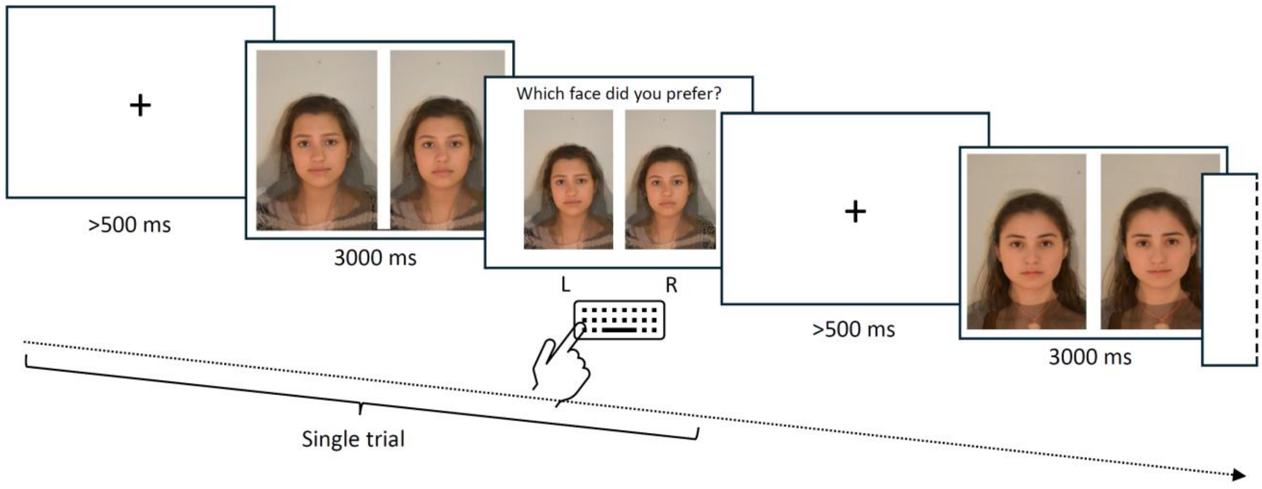

[8] Images were synthetic in the sense that none depicted a real person - they are averages across several actors (methodology outlined in Falconer et al., 2019)
9 Note, compassion expressions in the figure (left image in the first pair and right image in the second) are examples of 100% compassion expressions.

1.4. Procedure[10]

Session-1 was the first of two lab sessions. After consenting, the ECG electrodes were attached (Kamboj et al., 2023) and baseline (trait) and pre-stimulation (T1) state measures, were completed. The 30-min task-free stimulation period then commenced, after which participants completed the peri-stimulation (T2) state measures reflecting the task-free effects of active/sham stimulation, followed by Control-MIT or SC-MIT, during which, stimulation continued. The latter task-concurrent stimulation period therefore reflected the combined effects of stimulation and MIT. The final block of state measures (T3; post-MIT) were obtained straight after MIT. While stimulation continued, participants then completed the C-OMBAT and provided MIT adherence ratings. The stimulation device was then disconnected and participants provided with written instructions on correct use of the stimulator.

Session-8 (second lab session): The procedure for session-8 largely replicated session-1, except that attachment and childhood trauma questionnaires were omitted, and the Control-MIT /SC-MIT instructions were abbreviated, i.e. participants only listened to audio section iii (see above; (Kamboj et al., 2023)). In addition, at the end of this session participants provided posterior belief ratings on the effects of stimulation on their ability to form mental images. They also provided ratings on general and adverse stimulation-related sensations experienced during the preceding week.

Sessions 2-7 (at-home sessions): Despite the detailed instructions and demonstration provided on session-1, participants were required to follow on-screen instructions on attaching the stimulating electrodes and performing the thresholding procedure on each remote session. The study portal also presented participants' assigned MIT instructions (abbreviated section iii audio recordings). To minimize participant burden, only one block of state measures was taken on each remote session (corresponding to T3; post-MIT).

1.5. Statistical Analysis

1.5.1. Regression models

Regression diagnostics indicated that, except for HRV metrics (see below), the assumptions for linear modelling were met. The effects of stimulation and MIT on state measures over timepoints or sessions were therefore analyzed using linear mixed models (LMMs), with random intercepts, specifying one of two timeframes: *timepoint* on session 1 with three levels (T1-T3) or *session* with either two levels (sessions 1 and 8) *or* eight levels (sessions 1 *to* 8) depending on the outcome. For example, oculomotor-attentional bias (C-OMBAT) was only assessed once on each lab session, at the post-MIT timepoint (T3). Therefore, the only relevant time factor for this analysis was 'session' with two levels (session-1 and 8). Two levels of the session factor were also relevant for the analyses of between-session, cumulative/sustained HRV effects.

Because residuals from LMMs of HF-power (ms^2) and root mean square of successive differences in interbeat intervals (RMSSD) were seriously skewed we employed generalized LMMs (GLMMs) to examine time-dependent Stimulation x MIT effects for this data. These used a gamma distribution with a log-link function. As noted in the main paper, rapid/acute changes (i.e. timepoint effects) in

---

[10] Additional procedural details are provided in Kamboj et al (2023).

HRV were analysed based on interbeat interval (RR) data sampled in three 5-min epochs corresponding to T1-T3 on session-1 (Fig. 1, main paper). Cumulative Stimulation x MIT effects across sessions were based on RR data sampled at T1 (pre-stimulation) on session-1 and T3 (post-MIT) on session-8 values.

1.5.2. Probing interactions

Interactions were interpreted through visualization. In addition, significant higher-order (e.g. three-way) interactions were probed at lower levels (e.g. two-way and simple effects). As such, significant three-way interactions were decomposed into two, two-way analyses at each level of a third variable. For example, a significant Timepoint x Stimulation x MIT interaction would suggest that the effects of MIT over timepoints varied by stimulation condition (or vice versa). Therefore, two-way Timepoint x MIT interactions were examined separately for sham and tVNS to determine which of the two MIT conditions behaved differently in response to tVNS and/or Sham. Significant two-way interactions in these analyses were then further probed in simple effects analyses (e.g. following-up a significant Timepoint x MIT interaction in the tVNS stimulation condition by examining the simple effects (of timepoint) in each of the MIT conditions).

Selected pairwise tests *within* a particular condition (i.e. one of the four groups or one level of one of the two experimental factors) were then employed to evaluate specific hypotheses relating to acute/rapid *changes* in outcomes between pairs of timepoints (T1 v T2, T2, v T3, T1 v T3) in individual conditions. Depending on which condition was being tested (e.g. levels of a specific factor e.g. SC-MIT averaged across tVNS conditions or a specific Stimulation x MIT condition, e.g. tVNS+SC-MIT), each pair of timepoint comparisons was potentially uniquely informative. For example, *(i)* state self-compassion in the tVNS+SC-MIT group at T1 vs T2 should reflect the *isolated effect* of tVNS on this outcome, separate from any effect of SC-MIT. On the other hand, *(ii)* the T2 vs T3 comparison *isolates the effect* of SC-MIT in the sham + SC-MIT group (assuming a truly null effect of sham stimulation) or alternatively *(iii)* the T2 vs T3 comparison in the tVNS+SC-MIT group reflects the *additional* effect of SC-MIT on top of any effects of tVNS on self-compassion. Finally *(iv)* T1 v T3 tells us about the *combined effects* of both tVNS *and* SC-MIT, in the tVNS+SC-MIT group. Thus, where justified, specific informative hypotheses were tested with selected pairwise comparisons rather than a standard post hoc approach that tests all pairs of data points. In all such cases, effect sizes are reported to aid interpretation. Reported effect sizes relate to within-groups comparisons between pairs of timepoints or sessions ($d_{(Within)}$), or between groups comparisons ($d_{(Between)}$). These were calculated from model-derived estimated marginal means and standard errors, incorporating Pearson's *r* between repeated measures (Lenhard & Lenhard, 2022).

Between-condition comparisons were restricted to (i) T3 on Session-1 and (ii) the final data point on session-8. These compared tVNS+SC-MIT with the average of the three other conditions. All figures show model-derived estimated marginal means and standard errors, which also formed the basis for pairwise tests (Garofalo et al., 2022). Elsewhere (in text and tables), values are frequencies (and %) or means ± SD.

1.5.3. *Post hoc (non-preregistered) Bayesian analysis of HRV data*

After completing the current study we became aware of a large body of research summarized by Wolf et al (2021) showing strong support for the null hypothesis in studies of tVNS and HRV. We therefore performed post hoc independent samples Bayesian *t tests* of our HRV data in JASP (version 0.19.3). The dependent variables were HRV (RMSSD and HF power) *reactivity* (namely the change in HRV between T1 and T3 and Session 1 and 8).

2. SUPPLEMENTARY RESULTS

2.1. Additional Participant Characteristics

A *c*omprehensive description of the most relevant baseline/trait measures is provided in Table 1 of the main paper. Additional baseline trait measures are outlined in Table S2. The values in Table 1 (main paper) and Table S2 (below) align closely with normative or validation data from non-clinical samples (Collins, 1996) (Gilbert et al., 2011) (Gu et al., 2016) (Nunan et al., 2010) (Sinclair et al., 2012).

Table S2: Additional 'trait' measures. Values are Means ± SD.

	Sham + Con-MIT (n=30)	Sham + SC-MIT (n=30)	tVNS + Con-MIT (n=30)	tVNS + SC-MIT (n=30)
CTQ (Total)	39.17 (11.38)	37.40 (12.58)	38.40 (11.90)	40.30 (11.73)
AAS (Close)	3.32 (0.83)	3.68 (0.85)	3.62 (0.97)	3.47 (0.89)
AAS (Depend)	3.10 (0.91)	3.31 (0.88)	3.19 (0.91)	3.02 (0.92)
AAS (Anxiety)	2.64 (1.11)	2.50 (0.96)	2.76 (1.08)	2.98 (0.96)

CTQ: Childhood Trauma Questionnaire (short form; (Bernstein et al., 2003)
AAS: Adult Attachment Scale (revised, close relationship version (Collins, 1996)
Con-MIT=Control Mental Imagery Training; SC-MIT=Self-compassion-Mental Imagery Training

Because hormonal changes and menstrual phase are relevant to emotion regulation, we asked female participants about their use of hormonal contraceptive and their last period. Fifteen percent (n=13) of female participants used a hormone-based contraceptive. Of the remaining n=75 females (not using a hormonal contraceptive), the average number of days since menses was ~17 days. Days since menses was not, however, associated with any outcome.

2.2. Manipulation Checks

2.2.1. Credibility/Expectancy and Posterior Beliefs About tVNS and Mental Imagery Training
Because the three credibility/expectancy items (Table S3) were internally consistent (α=0.74), a single average was used to compare the groups. This showed no significant difference between conditions ($F(3,116)=0.32$, $p=0.811$). Table S3 also shows the posterior belief ratings of perceived improvement in mental imagery ability attributed to stimulation. These ratings were close to 0 (on a -4 to +4 scale) meaning that on average participants were relatively neutral in their beliefs (neither agreeing nor disagreeing) about the effects of stimulation on MIT; the groups did not differ in perceived improvement ratings at the end of the study ($F(3,116)=1.98$, $p=0.121$). Overall, this pattern of results suggests it is unlikely that these beliefs affected the pattern of effects seen with tVNS.

Table S3 – Credibility, expectancy and perceived improvement across the Stimulation x MIT conditions. Values are *M* ± *SD*

	Sham + Con-MIT (n=30)	Sham + SC-MIT (n=30)	tVNS + Con-MIT (n=30)	tVNS + SC-MIT (n=30)
Expectancy/credibility*				
Logical	5.63 (1.38)	5.77 (1.59)	5.77 (1.81)	5.57 (1.46)
Success expectancy	4.03 (1.33)	4.50 (1.50)	4.40 (1.63)	4.33 (1.35)
Would consider using	5.00 (1.80)	5.30 (1.54)	5.17 (1.82)	5.53 (1.80)
Posterior belief ⁋				
Perceived improvement	0.10 (1.71)	0.73 (1.70)	-0.33 (1.94)	0.30 (1.54)

Con-MIT=Control Mental Imagery Training; SC-MIT=Self-compassion-Mental Imagery Training
*Expectancy/credibility questions (session-1, after rationale for combining tVNS with mental imagery training, but prior to any MIT practice):
How logical does this approach of using the electrical vagus nerve stimulation to improve mental imagery seem to you?
How successful do you think this approach of electrical stimulation of the vagus nerve will be in changing your mental imagery abilities?
How seriously would you consider using this type of stimulation technique to improve your mental imagery abilities?
Responses rescaled to 0=Not at all to 8=Very.
⁋Perceived improvement question (session-8, after final assessments):
How much do you believe the following statement to be true? The stimulation improved my abilities to form mental images while listening to the audio instructions: +4=agree very much, 0=neither agree nor disagree, -4 'disagree very much'

2.2.2. Stimulation Parameters

Stimulation levels selected by participants increased across sessions ($\chi^2(7)=26.34$, $p<0.001$) but remained within a relatively narrow range (~13-15 mA; Fig.S3A) and did not differ between stimulation conditions (sham: *M*=13.59, *SD*=5.03 mA; tVNS: *M*=14.52, *SD*=4.58 mA averaged across sessions; $\chi^2(1)=1.14$, $p=0.287$) or show session-dependent differences between stimulation conditions (Session x Stimulation: $\chi^2(7)=4.73$, $p=0.693$).

The duration of stimulation was fixed during sessions 1 and 8 (the lab sessions) but could potentially vary between 0 and 30 min during the at-home self-administration sessions (depending on adherence). Based on the duration of participants' interactions with the stimulation instruction/countdown page on the study website, high levels of adherence were achieved. These did not differ across sessions (main effect of session: $\chi^2(5)=3.36$, $p=0.645$) or stimulation conditions (main effect of stimulation condition: $\chi^2(1)=0.12$, $p=0.730$; Fig.S3B) or vary across sessions in the two stimulation conditions (Session x Stimulation: $\chi^2(5)=2.63$, $p=0.756$). As shown in Fig. S3B, the recorded 'on-page' duration was consistent with the expected duration of stimulation (~30 min) for each at-home session.

Figure S3. Stimulation intensity and duration. **A**: Stimulation intensity (mA) by session in the tVNS and sham stimulation conditions (averaged across MIT conditions); **B**: Duration of stimulation (minutes) for the at-home stimulation sessions (session 2-7), based on how long participants spent on the stimulation instructions page on the study website. Values are *mean* ± *SE.*

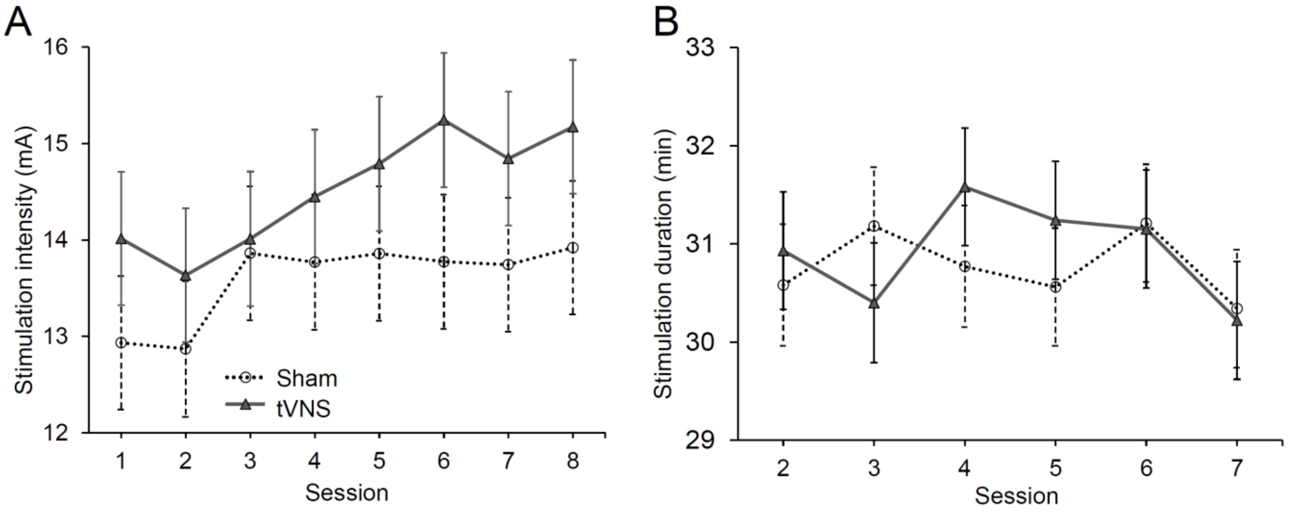

2.2.3. Adherence and Treatment Fidelity
Based on webpage data, 94% of participants remained on the stimulation countdown page for the full duration (~30-min) on ≥5 of the six remote stimulation sessions (sessions 2-7). This corresponded well with self-reported number of days of compliance with stimulator use (≥5 days: 96% across groups; *Fisher's exact test p*=0.166; Table S4). Based on participants interactions with the MIT audio instructions page of the website, 89% of participants were adherent to MIT instructions at home[11]. Again, this corresponded well with self-reported number of days on which participants indicated listening to the audio for their assigned MIT condition (≥5 days: 90% across groups; *Fisher's exact test p*=0.361; Table S4).

Relatedly, participants indicated high levels of fidelity to MIT instructions (i.e. ratings of how closely they followed the instructions). Specifically, they reported following the instructions relatively closely during MIT practices (Control-MIT: *M*=5.83, *SD*=1.23; SC-MIT: *M*=5.65, *SD*=1.22 on a 0-8 scale: 0=*did not follow instructions at all*; 8=*followed instructions very closely*) and did not differ between conditions (*p*=0.422; Fig. S4).

Table S4 – **Self-reported adherence with stimulation and MIT instructions.** Values are numbers of participants (%) who indicated completing ≥5 sessions of stimulation and MIT.

	Sham + Con-MIT (n=30)	Sham + SC-MIT (n=30)	tVNS + Con-MIT (n=30)	tVNS + SC-MIT (n=30)
Stimulation ≥5 sessions	27 (90%)	30 (100%)	28 (93%)	30 (100%)
MIT ≥5 sessions	25 (83%)	28 (93%)	26 (87%)	29 (97%)

Con-MIT=Control Mental Imagery Training; SC-MIT=Self-compassion-Mental Imagery Training.

[11] Participants listening to ≥50% the audio instructions for a particular session for sessions 2-7 (i.e. remained on the audio instruction page for ≥50% of the full duration) were deemed to be adherent with the MIT protocol for that session.

Figure S4: Mean (±SE) self-reported compliance with SC-MIT and Control-MIT instructions across sessions (averaged across stimulation conditions). Top and middle anchors provide reference points illustrating high and moderate self-reported adherence/fidelity(0= Did not follow the instructions at all).

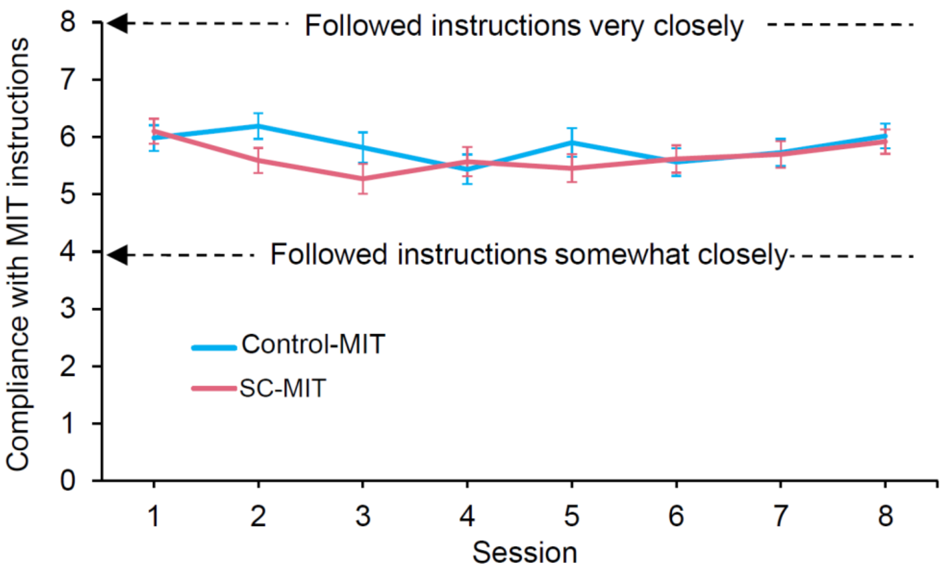

2.2.4. Specificity of Control Mental Imagery Training (Control-MIT): Effects on Vividness of Facial Imagery Questionnaire

To complement our demonstration of specificity of SC-MIT's effect on state self-compassion (Figure 2A and B, main paper), we examined whether there was a larger increase in facial mental imagery vividness in the Control-MIT condition relative to SC-MIT. Across the three timepoints on session-1, VFIQ scores showed a significant Timepoint x MIT interaction ($\chi^2(2)$=10.54,p=0.005; Figure S5A), but no interactions involving stimulation ($p\geq0.361$). Simple effects analyses showed a significant timepoint effect in the Control-MIT condition ($\chi^2(2)$=18.02, $p<0.001$), but not SC-MIT ($\chi^2(2)$=3.69,p=0.158). Pairwise comparisons between timepoints in Control-MIT showed a significant increase in vividness ratings for the target face between T2 (peri-stimulation) to T3 (post-MIT; $p<0.001$, d=0.54; Figure S5A).

There was also a differential effect of MIT condition on vividness of the target face across sessions (Session x MIT interaction: $\chi^2(7)$=25.15, p<0.001; Figure S5B), but no session-dependent interactions involving stimulation ($p\geq0.248$). Of note, in contrast to the within-session *increase* in vividness in the Control-MIT condition (collapsed across stimulation; Fig. S5A), facial imagery vividness for the target face (VFIQ-target face item) appeared to be *maintained* across sessions in the Control-MIT condition, with little fluctuation from session-1 to 8 (no simple effect of session: $\chi^2(7)$=9.60, p=0.212), whereas vividness *decreased* in the SC-MIT conditions (collapsed across stimulation; Fig. S5B), consistent with a lack of practice with this imagery type/forgetting of the target face ($\chi^2(7)$=65.03, p<0.001). The MIT conditions differed significantly in target face vividness at T3 (p=0.006) and at the last assessment on session-8($p<0.001$). Taken together with the specific effects of SC-MIT on state self-compassion and mindfulness (main paper), these findings confirm that participants in the two MIT conditions responded preferentially to their assigned MIT.

Figure S5: Vividness of Facial Imagery Questionnaire (VFIQ)-target face item in the SC-MIT and Control-MIT conditions (averaged across stimulation conditions). (A) Timepoint effects for the two MIT conditions. (B) Session effects. *Mean* ± *SE.* *** *p*<0.001; ***p*<0.01

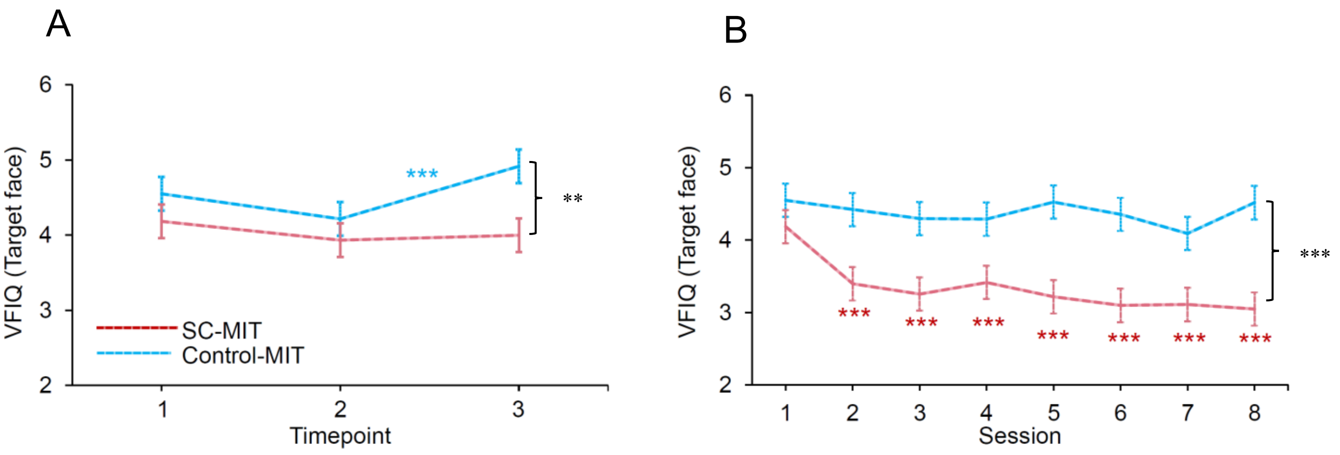

2.2.5. MIT Effects on General Mental Imagery Characteristics

Average ease of forming mental images (Control-MIT: 5.38 ± 1.64 *versus* SC-MIT: 5.99 ± 1.46) and clarity (Control-MIT: 5.66 ± 1.46 *versus* SC-MIT: 6.31 ± 1.20) were slightly higher in the SC-MIT condition ($p \geq 0.054$). However, valence (-4 to +4 scale) was significantly more positive in the SC-MIT condition (1.86 ± 0.93) than Control-MIT (0.52 ± 0.97; $p<0.001$)

2.2.6. General Stimulation-related Sensations and Adverse Effects

All participants endorsed the "tingling" item, with a mean rating >4 on a 0-8 scale (indicating at least 'moderate' tingling sensation; Table S5). The majority also endorsed the "pulsing" (n=115; 96%) and "discomfort" (n=108; 90%) items, although these were rated <4 on average (Table S5). Ratings on these scales did not differ between stimulation conditions (*p* values ≥0.083).

The frequency of occurrence of adverse effects (*any* degree of intensity >0 on ear pain, skin irritation, headache, dizziness, face pain, upset stomach, cough, common cold symptoms) attributed by participants to stimulation are outlined in Table S5. The most frequently endorsed adverse effect was 'ear pain' (74.2% scored >0 out of 8), although its occurrence did not differ between conditions ($\chi^2(1)=0.391$, $p=0.532$). However, although ratings of *intensity* of ear pain were generally low, they were significantly higher with tVNS ($M=2.60 \pm 2.26$ on a 0-8 scale) than sham ($M=1.72 \pm 1.62$; $t(118)=2.47$, $p=0.015$). There was no difference in occurrence of any other pre-specified adverse effect (≤20% across conditions; $\chi^2 \leq 2.828$; *p* values ≥0.093; Table S5). Other than the eight pre-specified adverse effects (Table S5), a minority of participants (n=18) indicated one or two additional (non pre-specified) adverse effects of stimulation. These included "sleepiness" or "drowsiness" (sham: n=3, tVNS: n=2) and "itching" (sham: n=1; tVNS: n=2) which were the two most common. Others (nausea, numbness of the stimulation site, mental fog, neck pain, dry mouth, eyelid twitching, facial tingling, low mood, irritation and anxiety) were each noted by n=1 and fatigue by n=2. The number of participants indicating these additional adverse effects was matched between stimulation conditions (n=9 in each)

Table S5: Protocol-specified general and adverse effects of stimulation.

	Sham (n=60)	tVNS (n=60)
*General sensation intensity**	*Mean* (*SD*)	*Mean* (*SD*)
Tingling	4.67 (1.04)	5.03 (1.25)
Pulsing	3.95 (1.47)	3.75 (1.61)
Discomfort	2.58 (1.69)	3.02 (1.85)
*Adverse effects: occurrences**	*n* (%)	*n* (%)
Ear pain	43 (71.7%)	46 (76.7%)
Skin irritation	12 (20.0%)	12 (20.0%)
Headache	11 (18.3%)	12 (20.0%)
Dizziness	14 (23.3%)	7 (11.7%)
Face pain	6 (10.0%)	9 (15.0%)
Upset stomach	7 (11.7%)	6 (10.0%)
Cough	7 (11.7%)	4 (6.7%)
Common cold symptoms	5 (8.3%)	5 (8.3%)

Note, all effects (general and adverse) were scored on a 9-point severity range (0="not at all"*; 4=*"moderate"*; 8="*severe"*). Because of the infrequent occurrence of adverse effects (except ear pain), these responses were dichotomized (0=absent; >0=present). '*Occurrence'* therefore refers to any score >0 on the adverse events items.

2.3. Further Details on Analysis of the Primary and Secondary Outcomes

2.3.1. State and Trait Self-compassion

A significant three-way interaction was found acutely on state self-compassion, as reported in the main paper. Table S6A, below, shows the test statistics associated with the seven estimated parameters (one three-way and three two-way interactions and three main effects), and the follow-up analyses designed to 'isolate' the source of the interaction (two-way analyses and simple effects) prior to pairwise tests reported in the main paper. Cumulative effects were similarly tested in a three-way analysis, yielding only an interaction between timepoint and MIT condition (Table S6B), which was probed using simple effects. The three way analysis on trait self-compassion (Table S6C) showed a Timepoint x Stimulation interaction which is visualised in the main paper, with no further statistical decomposition.

Table S6. State and trait self-compassion regression models (test statistics). A. *Rapid/acute effects of Stimulation + MIT across timepoints on session-1 on state self-compassion.* The main interactions of interest (Timepoint x Stimulation, Timepoint x MIT and Timepoint x Stimulation x MIT) are in bold. The significant three-way analysis was decomposed into two, two-way Timepoint x Stimulation analyses at each level of the MIT factor (i.e. separately for SC-MIT and Control-MIT); the significant two-way analysis in the SC-MIT condition was further probed in simple effects analyses in each of the SC-MIT groups: tVNS_SC-MIT and Control-MIT. **B.** *Cumulative/sustained effects across daily sessions (state self-compassion).* The three-way analysis only yielded a significant two-way Session x MIT interaction which was probed using simple effects in SC-MIT and Control-MIT. **C.** *Cumulative /sustained effects on session-1 and 8 (trait self-compassion measure).*

A.

3-WAY ANALYSIS (FULL MODEL)

State Self-compassion (rapid/acute)	*df*	χ^2	*p*
Timepoint	2	34.04	<0.001
Stimulation	1	1.33	0.249
**Timepoint x Stimulation**	**2**	**4.99**	**0.083**
MIT	1	0.45	0.504
**Timepoint x MIT**	**2**	**16.47**	**<0.001**
Stimulation x MIT	1	0.66	0.417
**Timepoint x Stimulation x MIT**	**2**	**7.4**	**0.025**

2-WAY

**SC-MIT**	*df*	χ^2	*p*
Timepoint	2	43.43	<0.001
Stimulation	1	2.10	0.148
Timepoint x Stimulation	2	12.14	0.0023

**Control-MIT**	*df*	χ^2	*p*
Timepoint	2	9.26	0.01
Stimulation	1	0.05	0.817
**Timepoint x Stimulation**	**2**	**0.93**	**0.628**

SIMPLE EFFECTS

**tVNS+SC-MIT**	*df*	χ^2	*p*
**Timepoint**	**2**	**51.69**	**<0.001**

**Sham+SC-MIT**	*df*	χ^2	*p*
Timepoint	2	6.77	0.034

B.

3-WAY ANALYSIS (FULL MODEL)

State Self-compassion (cumulative/sustained)	*df*	χ^2	*p*
Session	7	79.50	<0.001
Stimulation	1	0.16	0.687
**Session x Stimulation**	**7**	**5.83**	**0.560**
MIT	1	8.45	0.004
**Session x MIT**	**7**	**58.73**	**<0.001**
Stimulation x MIT	1	0.09	0.766
**Session x Stimulation x MIT**	**7**	**2.06**	**0.956**

Simple Effects

**SC-MIT**	*df*	χ^2	*p*
**Session**	**7**	**133.43**	**<0.001**

**Control-MIT**	*df*	χ^2	*p*
Session	7	2.85	0.899

C

3-WAY ANALYSIS (FULL MODEL)

Trait self-compassion (cumulative/sustained)	*df*	χ^2	*p*
Session	1	0.25	0.6141
Stimulation	1	0.28	0.5977
**Session x Stimulation**	**1**	**4.16**	**0.0415**
MIT	1	0.75	0.3879
**Session x MIT**	**1**	**0.05**	**0.8265**
Stimulation x MIT	1	0.12	0.7298
**Session x Stimulation x MIT**	**1**	**0.62**	**0.4299**

2.3.2. Self-criticism

As outlined in the main paper, self-criticism showed time-dependent effects of MIT condition both acutely (Timepoint x MIT, $\chi^2(2)=15.16$, $p<0.001$; Fig. S6A; Table S7A), and between sessions (Session x MIT, $\chi^2(7)=24.43$, $p=0.001$;Fig. S6B; Table 7B).

Considering the rapid/acute effects first, simple effects of timepoint were found in the Control-MIT ($\chi^2(2)= 61.24$, $p<0.001$) and SC-MIT conditions ($\chi^2(2)= 100.29$, $p<0.001$; Table S7A). However, the *size* of the change (decrease) in self-criticism clearly differed across timepoints in the two conditions (Figure S6A). Although the T1→T2 slopes were virtually identical (Control-MIT: $d_{(Within)}=-1.1$, $p<0.001$,; SC-MIT: $d_{(Within)}=-1.0$, $p<0.001$; Fig S6A), the T2→T3 slope - which represent the *additional* effect of MIT (on top of non-specific effects of timepoint) on self-criticism - was flat in the Control-MIT condition ($d_{(Within)}<0.1$, $p>0.99$) but showed a further large decline in the SC-MIT condition ($d_{(Within)}=-1.2$, $p<0.001$). The difference between SC-MIT and Control-MIT at T3 was small-moderate and significant ($p=0.034$, $d_{(Between)}=0.39$)

Between-session changes in self-criticism are displayed in Figure S6B. Simple effects analyses showed significant session effects in both the Control-MIT ($\chi^2(7)= 148.5$, $p<0.001$) and the SC-MIT condition ($\chi^2(7)= 343.48$, $p<0.001$). Pairwise comparisons showed significant differences between session-1 and each subsequent session in both MIT conditions (all *p* values<0.001), although effect sizes were larger in SC-MIT (e.g. session-1 *versus* session-2, $d=-1.8$; session-1 *versus* session-8: $d=-2.0$) relative to Control-MIT (e.g. session-1 *versus* session-2, $d=-0.9$; session-1 *versus* session-8: $d=-1.3$). The difference between MIT conditions at the final assessment (session 8) was moderate in size and significant ($p=0.016$, $d_{(Between)}=0.45$).

Figure S6: Mental Imagery Training (MIT) effects across time (timepoint and session) A: Acute MIT effects (collapsed across stimulation condition) across timepoints (T1-T3). B: Session x MIT effects. ****p*<0.001: pairwise for (A) T1 *v* T2 (SC-MIT and Control-MIT), T2 *v* T3 (SC-MIT), T1 *v* T3 (SC-MIT) and (B) session-1 *v* subsequent sessions (SC-MIT and Control-MIT). *p<0.05: between MIT conditions on T3 (session-1) and final assessment (session-8). Values are *means* ± *SE*.

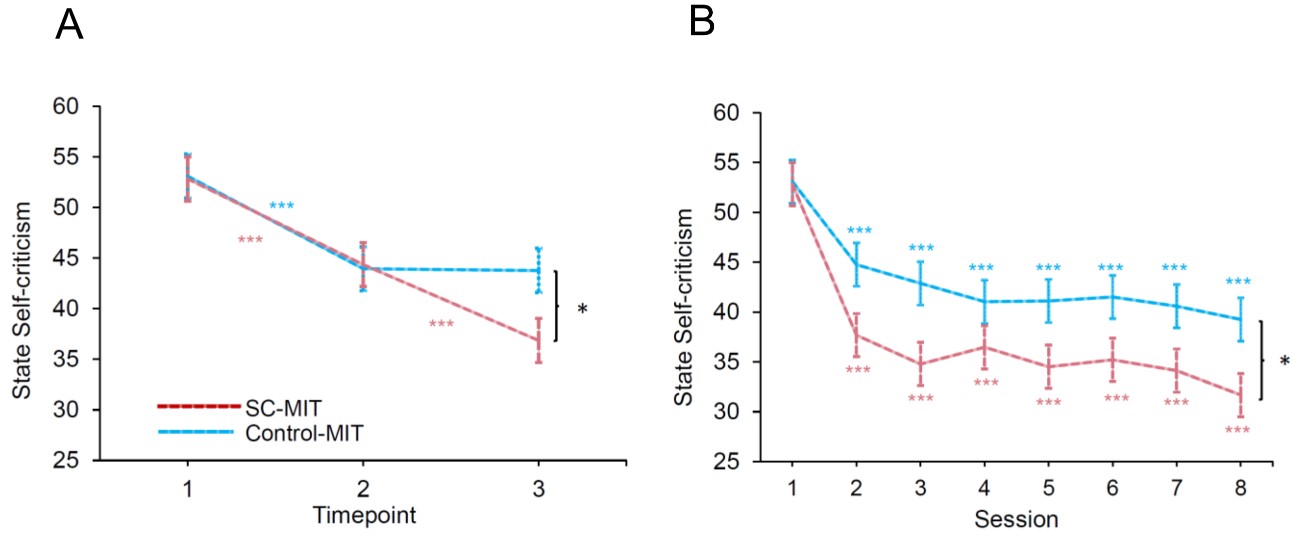

Table S7. State self-criticism regression models (test statistics). A. *Rapid/acute effects of Stimulation + MIT across Timepoints on Session-1.* The main interactions of interest (Timepoint x Stimulation, Timepoint x MIT and Timepoint x Stimulation x MIT) are in bold. The three-way model yielded a significant two-way interaction which was probed using simple effects analyses for SC-MIT and Control-MIT.
B. *Cumulative/sustained effects across daily sessions.* The three-way analysis yielded a significant two-way Session x MIT interaction which was probed using simple effects in SC-MIT and Control-MIT.

A

3-WAY ANALYSIS – FULL MODEL

State Self-criticism (rapid/acute)	*df*	*χ2*	*p*
Timepoint	2	155.3	<0.001
Stimulation	1	2.58	0.108
**Timepoint x Stimulation**	**2**	**2.60**	**0.273**
MIT	1	0.66	0.417
**Timepoint x MIT**	**2**	**15.16**	**<0.001**
Stimulation x MIT	1	<0.01	0.946
**Timepoint x Stimulation x MIT**	**2**	**1.44**	**0.487**

SIMPLE EFFECTS

**SC-MIT**	*df*	χ^2	*p*	**Control-MIT**	*df*	χ^2	*p*
**Timepoint**	**2**	**100.29**	**<0.001**	**Timepoint**	**2**	**61.24**	**<0.001**

B

3-WAY ANALYSIS – FULL MODEL

State Self-criticism(cumulative/sustained)	*df*	*χ2*	*p*
Session	7	469.89	<0.001
Stimulation	1	5.06	0.0245
**Session x Stimulation**	**7**	**1.92**	**0.9642**
MIT	1	4.59	0.0322
Session x MIT	7	24.43	0.001
Stimulation x MIT	1	0.01	0.9287
**Session x Stimulation x MIT**	**7**	**6.45**	**0.4878**

SIMPLE EFFECTS

**SC-MIT**	*df*	χ^2	*p*	**Control-MIT**	*df*	χ^2	*p*
**Session**	**7**	**343.5**	**<0.001**	**Session**	**7**	**148.6**	**<0.001**

2.3.3. HRV

2.3.3.1. Frequentist Analysis of HRV

As noted in the main paper, GLMMs of both RMSSD and HF-power showed increases across both timescales (acutely within session-1 and cumulatively from session-1 to 8). The time-dependent effects are shown in Fig. S7 and regression tests statistics in Table S8.

Figure S7: Time-(timepoint and session) dependent effects of stimulation and MIT condition on HRV metrics. A: RMSSD values at timepoints T1-T3. Main effect of timepoint is illustrated in the black line graph (averaged across the four Stimulation x MIT conditions); pale background line graphs represent the effects of time in the four separate groups (solid red: tVNS+SC-MIT; dashed red: sham+SC-MIT; sold blue: tVNS+ Control-MIT; dashed blue: sham+ Control-MIT). **B:** High frequency power (ms^2) across timepoints T1-T3. **C:** RMSSD on sessions 1 (T1; baseline) and 8 (T3, last measurement) collapsed across stimulation and MIT conditions. **D:** High frequency power on sessions 1 and 8. Pairwise tests: **p*<0.05 ****p*<0.001. Symbols represent *mean* ± *SE.*

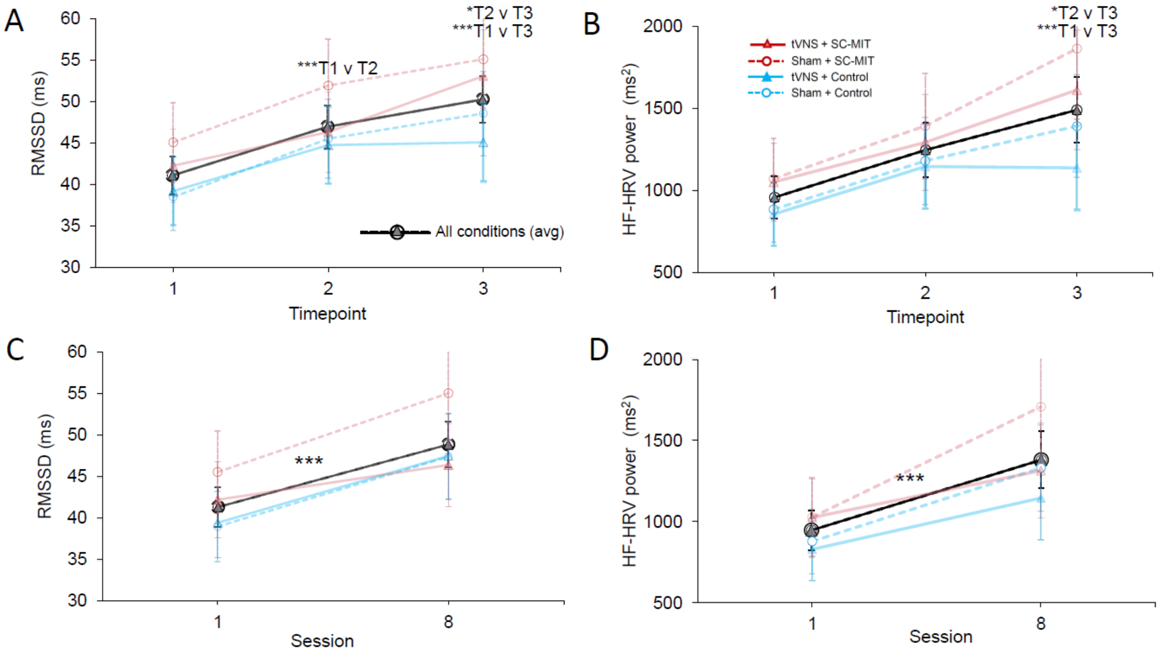

Table S8 summarizes the test statistics for the three-way and two-way interactions between, and main effects of, Time (timepoint or session), stimulation and MIT for the two pre-registered measures of HRV(RMSSD and HF-Power respectively). As can be seen, none of the substantive (two or three-way) interactions (Time (Timepoint or Session) x Stimulation x MIT) were significant. Only the trivial timepoint/session main effects illustrated in Fig. S7 A-D showed a significant effect.

Table S8: HRV – RMSSD: Rapid/acute effects of Stimulation + MIT across Timepoints on Session-1

A **3 WAY MODELS**

HRV-RMSSD (rapid/acute)	*df*	χ^2	*p*	*HRV-RMSSD (cumulative/sustn)*	*df*	χ^2	*p*
Timepoint	2	67.66	<0.001	Session	1	17.00	<0.001
Stimulation	1	0.24	0.628	Stimulation	1	0.35	0.554
**Timepoint x Stimulation**	**2**	**0.81**	**0.666**	**Session x Stimulation**	**1**	**0.42**	**0.518**
MIT	1	1.34	0.247	MIT	1	0.79	0.375
**Timepoint x MIT**	**2**	**1.54**	**0.463**	**Session x MIT**	**1**	**0.36**	**0.546**
Stimulation x MIT	1	0.06	0.810	Stimulation x MIT	1	0.44	0.509
**Timepoint x Stimulation x MIT**	**2**	**2.22**	**0.330**	**Session x Stimulation x MIT**	**1**	**0.28**	**0.597**

B **3 WAY MODELS**

HRV-HF-power (rapid/acute)	*df*	χ^2	*p*	*HRV-HF-power (cumulat/sustn)*	*df*	χ^2	*p*
Timepoint	2	58.38	<0.001	Session	1	20.92	<0.001
Stimulation	1	0.17	0.677	Stimulation	1	0.37	0.543
**Timepoint x Stimulation**	**2**	**1.91**	**0.385**	**Session x Stimulation**	**1**	**1.17**	**0.279**
MIT	1	1.20	0.273	MIT	1	0.96	0.328
**Timepoint x MIT**	**2**	**2.54**	**0.281**	**Session x MIT**	**1**	**<0.01**	**0.958**
Stimulation x MIT	1	0.00	0.984	Stimulation x MIT	1	0.01	0.942
**Timepoint x Stimulation x MIT**	**2**	**0.21**	**0.901**	**Session x Stimulation x MIT**	**1**	**0.26**	**0.608**

Controlling for potential confounds (age, sex, DASS-21 anxiety and depression, SOC-S trait compassion) did not alter the results of the HRV analyses ($p \geq 0.200$). Neither was there any indication that stimulation condition interacted with previously reported moderators of the effects of biological interventions and compassion training on HRV metrics (avoidant or dependent attachment; (Kamboj et al., 2015; Kamboj et al., 2018; Rockliff et al., 2011)).

2.3.3.2. Bayesian analyses of HRV

The lack of evidence for differential effects of stimulation on HRV metrics is consistent with a recent Bayesian meta-analysis of the effects of auricular tVNS on HRV. This showed strong evidence for the lack of an effect of stimulation on HRV (BF_{01}~25; (Wolf et al., 2021)). We therefore supplemented our frequentist analyses with (non-pre-registered) Bayesian analyses of our main HRV measures. For consistency with Wolf et al (2021) we report *between-groups* (tVNS *v* sham) Bayesian t-tests. Specifically, we compared the effects of tVNS *v* sham stimulation on acute (T1→T3) and sustained (Session-1→8) changes (i.e. *reactivity*) in RMSSD and HF-power. These provided moderately strong evidence for a lack of effect of stimulation on both pre-registered HRV metrics

and across both time periods. The comparison between stimulation conditions for acute ΔRMSSD gave BF_{01} =4.96, and for sustained ΔRMSSD, BF_{01} =3.32. Acute ΔHF-power gave BF_{01} =4.23, and BF_{01} =2.47 for sustained ΔHF-power.

The BF_{01} values were relatively unaffected when T1→T2 reactivity or topic HRV values at T2 or T3 were used as the dependent variable instead of T1→T3 (BF_{01}=4-5).

2.3.4. Negative and Positive Affect

2.3.4.1. PANAS-Negative

PANAS-negative scores at baseline (T1) were close to floor levels at baseline on session-1 (T1: *M*=6.83, *SD=2*.41 on a 5-25 scale; Fig S8A) and showed a further generalized reduction over T2 and T3 (timepoint main effect: $\chi^2(2)=42.02$, $p<0.001$. There were no effects of stimulation or MIT ($p\geq0.255$). Similarly, there was a generalized reduction in negative affect across sessions (main effect of session: $\chi^2(7)=53.49$; $p<0.001$; Figure S8B) but this was also not moderated by MIT or stimulation ($p\geq0.277$).

2.3.4.2. PANAS-Positive and TPAS-safe/warm

PANAS-positive scores also showed a generalized *reduction* across timepoints (T1 *M*=14.49, *SD*= 3.58 to T3 *M*=12.17, *SD*=4.17; Fig S8C) , reflecting a main effect of timepoint ($\chi^2(2)=51.63$, $p<0.001$) which again, was not moderated by MIT or stimulation condition ($p\geq0.407$). There was also a reduction in PANAS-positive across sessions (main effect of session: $\chi^2(7)=208.41$, $p<0.001$; Figure S8D) which was moderated by MIT condition (Session x MIT; $\chi^2(7)=15.21$, $p=0.033$) but not stimulation ($p\geq0.342$). Simple effects of session were significant in both MIT conditions (Control-MIT: $\chi^2(7)=112.461$, $p<0.001$; SC-MIT $\chi^2(7)=108.51$, $p<0.001$). All pairwise comparisons between session-1 and subsequent sessions: $p<0.001$. In contrast to PANAS-Positive, TPAS-safe/warm scores showed a generalised *upward* trend across timepoints ($\chi^2(2)=19.04$, $p<0.001$; Figure S8E). However, this was not moderated by stimulation or MIT ($p\geq0.135$). Relatedly, the increase in TPAS-safe/warm across sessions ($\chi^2(7)=20.24$, $p=0.005$; Figure S8F), was also not moderated by stimulation and/or MIT ($p\geq0.392$).

Figure S8: Timepoint and session effects on positive and negative affect. *Mean ± SE*

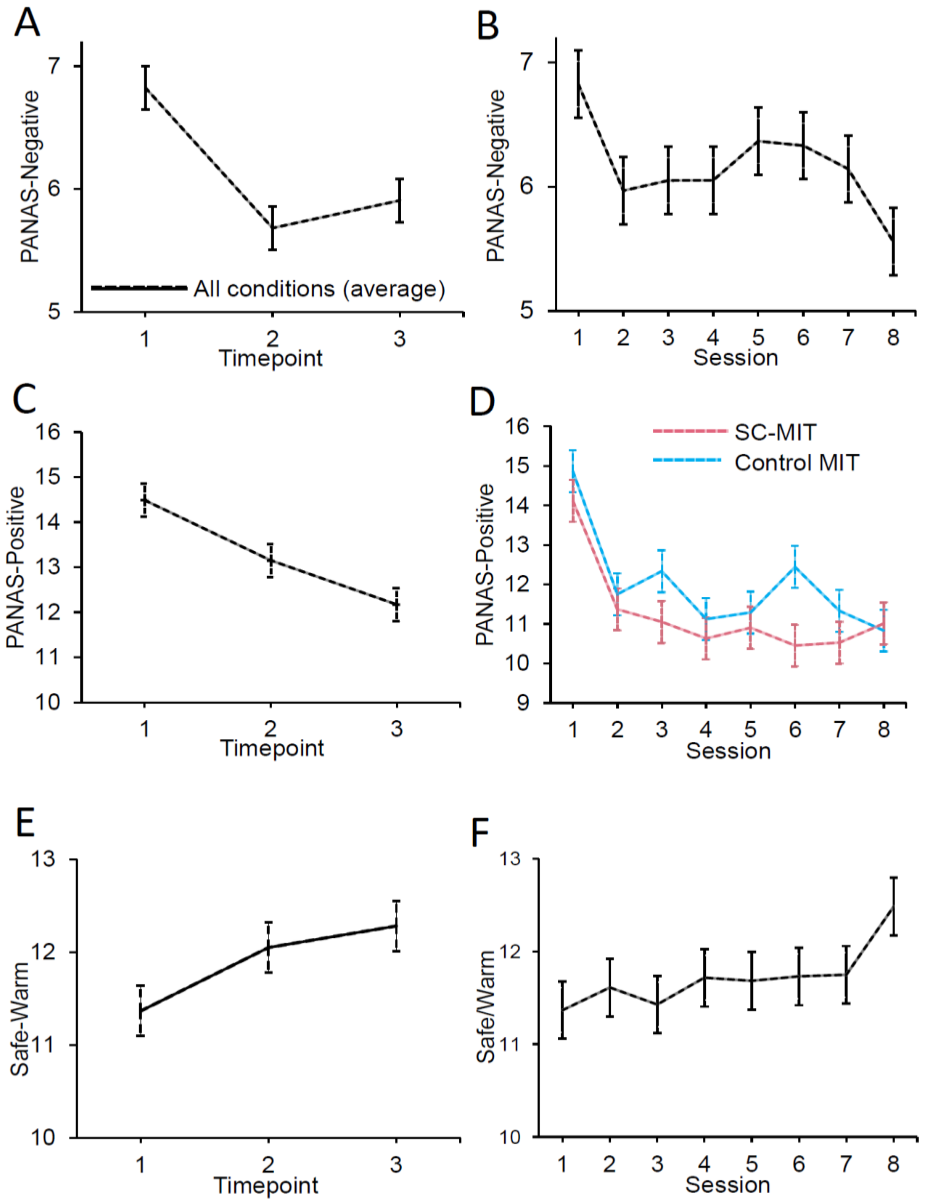

2.4. Oculomotor attentional bias (C-OMBAT) performance and trait self-compassion

Figure S9 shows the association between the change (session-1→8) in trait self-compassion (ΔSOCS-S) and Δpupil size in the two stimulation conditions. As described in the main text, the strength of association differed significantly between the two stimulation conditions (z=2.18, p=0.0293), reflecting a stronger positive correlation between ΔSOC-S and Δpupil size in the tVNS condition (r(56)=0.35, p=0.007; *cf.* Control-MIT: r(55)=-0.05, p=0.696).

Figure S9: Association between change in pupil size and trait self-compassion across session.
Association between ΔTrait self-compassion (ΔSOC-S) and ΔPupil size (z-transformed). NB: The y-axis is truncated between 25 and 55. A single outlier at ΔSOCS=54 is indicated with an arrow (this datapoint was *included* in the analyses described in the text, although effects were unaltered by removal).

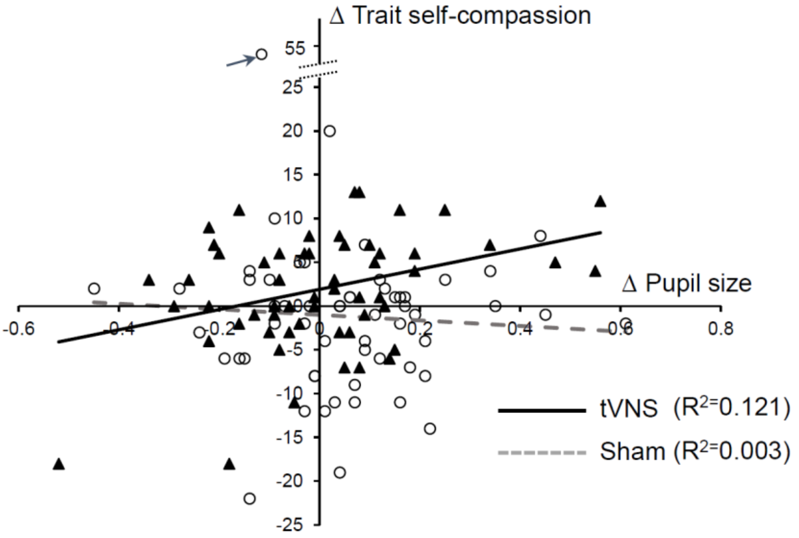

REFERENCES

Bernstein, D. P., Stein, J. A., Newcomb, M. D., Walker, E., Pogge, D., Ahluvalia, T., Stokes, J., Handelsman, L., Medrano, M., & Desmond, D. (2003). Development and validation of a brief screening version of the Childhood Trauma Questionnaire. *Child Abuse & Neglect*, *27*(2), 169-190. https://doi.org/doi:10.1016/S0145-2134(02)00541-0

Collins, N. L. (1996). Working models of attachment: Implications for explanation, emotion, and behavior. *Journal of Personality and Social Psychology*, *71*(4), 810.

Falconer, C. J., Lobmaier, J. S., Christoforou, M., Kamboj, S. K., King, J. A., Gilbert, P., & Brewin, C. R. (2019). Compassionate faces: Evidence for distinctive facial expressions associated with specific prosocial motivations. *PLoS One*, *14*(1), e0210283. https://doi.org/10.1371/journal.pone.0210283

Farmer, A. D., Strzelczyk, A., Finisguerra, A., Gourine, A. V., Gharabaghi, A., Hasan, A., Burger, A. M., Jaramillo, A. M., Mertens, A., & Majid, A. (2021). International consensus based review and recommendations for minimum reporting standards in research on transcutaneous vagus nerve stimulation (version 2020). *Frontiers in human neuroscience*, *14*, 568051. https://doi.org/10.3389/fnhum.2020.568051

Garofalo, S., Giovagnoli, S., Orsoni, M., Starita, F., & Benassi, M. (2022). Interaction effect: Are you doing the right thing? *PLoS One*, *17*(7), e0271668.

Gilbert, P. (2014). The origins and nature of compassion focused therapy. *British journal of clinical psychology*, *53*(1), 6-41. https://doi.org/10.1111/bjc.12043

Gilbert, P., McEwan, K., Matos, M., & Rivis, A. (2011). Fears of compassion: Development of three self-report measures. *Psychology and Psychotherapy: Theory, research and practice*, *84*(3), 239-255. https://doi.org/10.1348/147608310X526511

Gu, J., Strauss, C., Crane, C., Barnhofer, T., Karl, A., Cavanagh, K., & Kuyken, W. (2016). Examining the factor structure of the 39-item and 15-item versions of the Five Facet Mindfulness Questionnaire before and after mindfulness-based cognitive therapy for people with recurrent depression. *Psychological Assessment*, *28*(7), 791. https://doi.org/10.1037/pas0000263

Kamboj, S. K., Kilford, E. J., Minchin, S., Moss, A., Lawn, W., Das, R. K., Falconer, C. J., Gilbert, P., Curran, H. V., & Freeman, T. P. (2015). Recreational 3, 4-methylenedioxy-N-methylamphetamine (MDMA) or 'ecstasy'and self-focused compassion: preliminary steps in the development of a therapeutic psychopharmacology of contemplative practices. *Journal of Psychopharmacology*, *29*(9), 961-970. https://doi.org/10.1177/0269881115587143

Kamboj, S. K., Peniket, M., & Simeonov, L. (2023). A bioelectronic route to compassion: Rationale and study protocol for combining transcutaneous vagus nerve stimulation (tVNS) with compassionate mental imagery. *PLoS One*, *18*(3), e0282861. https://doi.org/ 10.1371/journal.pone.0282861

Kamboj, S. K., Walldén, Y. S., Falconer, C. J., Alotaibi, M. R., Blagbrough, I. S., Husbands, S. M., & Freeman, T. P. (2018). Additive effects of 3, 4-methylenedioxymethamphetamine (MDMA) and compassionate imagery on self-compassion in recreational users of ecstasy. *Mindfulness*, *9*(4), 1134-1145. https://doi.org/10.1007/s12671-017-0849-0

Kroenke, K., Spitzer, R. L., & Williams, J. B. (2003). The Patient Health Questionnaire-2: validity of a two-item depression screener. *Medical care*, 1284-1292.

Kroenke, K., Spitzer, R. L., Williams, J. B., Monahan, P. O., & Löwe, B. (2007). Anxiety disorders in primary care: prevalence, impairment, comorbidity, and detection. *Annals of internal medicine*, *146*(5), 317-325. https://doi.org/https://doi.org/10.7326/0003-4819-146-5-200703060-00004

Lenhard, W., & Lenhard, A. (2022). *Computation of effect sizes*. Retrieved 11th May 2024 from https://www.psychometrica.de/effect_size.html

Nunan, D., Sandercock, G. R., & Brodie, D. A. (2010). A quantitative systematic review of normal values for short-term heart rate variability in healthy adults. *Pacing and clinical electrophysiology*, *33*(11), 1407-1417.

Petrocchi, N., Di Bello, M., Cheli, S., & Ottaviani, C. (2022). Compassion focused therapy and the body: How physiological underpinnings of prosociality inform clinical practice. In G. S. Paul Gilbert (Ed.), *Compassion Focused Therapy: Clinical Practice and Application* (pp. 345-359). Routledge.

Porges, S. W. (2017). Vagal pathways: portals to compassion. In E. S.-T. Emma M. Seppälä, Stephanie L. Brown, Monica C. Worline, C. Daryl Cameron, James R. Doty (Ed.), *The Oxford handbook of compassion science* (pp. 189-202). Oxford University Press.

Rockliff, H., Karl, A., McEwan, K., Gilbert, J., Matos, M., & Gilbert, P. (2011). Effects of intranasal oxytocin on'compassion focused imagery'. *Emotion*, *11*(6), 1388.

Sinclair, S. J., Siefert, C. J., Slavin-Mulford, J. M., Stein, M. B., Renna, M., & Blais, M. A. (2012). Psychometric evaluation and normative data for the depression, anxiety, and stress scales-21 (DASS-21) in a nonclinical sample of US adults. *Evaluation & the health professions*, *35*(3), 259-279.

Stellar, J. E., & Keltner, D. (2017). Compassion in the autonomic nervous system: The role of the vagus nerve. In P. Gilbert (Ed.), *Compassion: Concepts, Research and Applications* (pp. 120-134). Routledge.

Wolf, V., Kühnel, A., Teckentrup, V., Koenig, J., & Kroemer, N. B. (2021). Does transcutaneous auricular vagus nerve stimulation affect vagally mediated heart rate variability? A living and interactive Bayesian meta-analysis. *Psychophysiology*, *58*(11), e13933. https://doi.org/10.1111/psyp.13933
